# Serendipitous *N*,*S*-difunctionalization of triazoles with trifluoromethyl-β-diketones: access to regioisomeric 1-trifluoroacetyl-3-aryl-5-(2-oxo-2-arylethylthio)-1,2,4-triazoles as DNA-groove binders†

**DOI:** 10.1039/d4ra00083h

**Published:** 2024-02-23

**Authors:** Ranjana Aggarwal, Prince Kumar, Mona Hooda, Suresh Kumar

**Affiliations:** a Department of Chemistry, Kurukshetra University Kurukshetra-136119 Haryana India; b Council of Scientific and Industrial Research-National Institute of Science Communication and Policy Research New Delhi 110012 India ranjana67in@yahoo.com ranjanaaggarwal67@gmail.com +91-9896740740; c Department of Chemistry, Gurugram University Gurugram-122003 Haryana India

## Abstract

In the present research work, a serendipitous regioselective synthesis of DNA targeting agents, 1-trifluoroacetyl-3-aryl-5-(2-oxo-2-arylethylthio)-1,2,4-triazoles, has been achieved through the one-pot cascade reaction of 3-mercapto[1,2,4]triazoles with trifluoromethyl-β-diktetones in presence of NBS instead of the cyclized thiazolo[3,2-*b*][1,2,4]triazole. The present protocol offered a unique approach for functionalizing both *N*-acylation and *S*-alkylation in a concerted fashion. The structures of the regioisomeric products were thoroughly characterized by heteronuclear 2D NMR experiments. Facile scalability and excellent atom economy through easily available starting reactants are the notable features of the present sustainable protocol. Targeting tumor cell DNA with minor groove-binding small molecules has proven highly effective in the recent past, drawing significant attention for combating tumor-related afflictions. In this context, the synthesized analogs were primarily screened for their ability to bind with the DNA duplex d(CGCGAATTCGCG)_2_ using molecular modeling tools. Additionally, the most promising compound 14m was deployed as a probe for DNA sensing and interaction mechanisms with calf thymus (ct)DNA through various spectral techniques at a physiologic temperature of 37 °C. It has been found that the compound demonstrated a strong binding affinity (*K*_b_ = 1 × 10^5^ M^−1^) with double-helical DNA, particularly within the minor groove, resulting in the formation of a stable complex through static quenching (*K*_q_ = 5.86 ± 0.11 × 10^12^ M^−1^ s^−1^). The fluorescent displacement assay confirmed that the quencher binds to the minor groove of ctDNA, further supported by circular dichroism and viscosity studies.

## Abstract 2

An expedient construction of DNA groove binder; CF_3_-containing triazoles *via* regioselective reaction of trifluoromethyl-β-diketones with 3-mercapto[1,2,4]triazoles.

## Graphical abstract

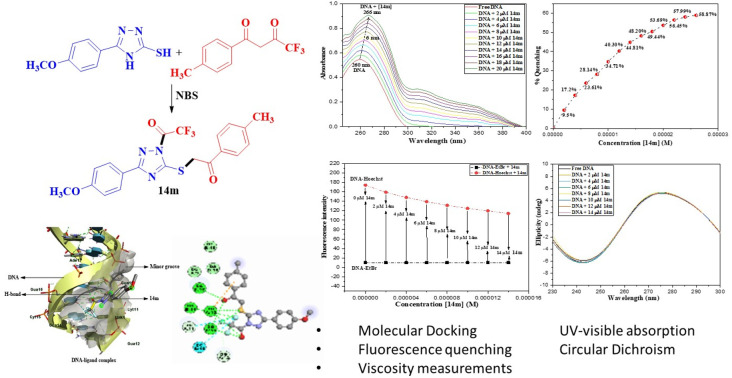

## Introduction

Organofluorine chemistry irrefutably holds immense importance in the realm of scientific exploration, particularly in the pharmaceutical and agrochemical industries, and makes up more than 50% of the best-selling drug molecules approved by the US Food and Drug Administration (FDA).^1^ Within the category of organofluorine compounds, those containing the trifluoromethyl (CF_3_) group are particularly noteworthy due to their potent electron-withdrawing properties and considerable hydrophobic surface area. The inclusion of the CF_3_ group in an organic compound can significantly alter a plethora of physiochemical characteristics, leading to improved effectiveness, specificity, ability to dissolve in fats, ability to be absorbed by the body, resistance to metabolism, electrical charge, ability to pass through membranes, polarity, and durability of C–F bond compared to C–H bond.^2,3^ Trifluoromethyl group is utilized as a bioisostere for chlorine, methyl, or nitro groups in the development of new medications.^4–6^ Indeed, numerous successful drugs have capitalized on the benefits of incorporating the trifluoromethyl group into their chemical makeup. It has been well-documented that the linkage of CF_3_ groups to various heterocyclic molecules renders them highly effective as DNA targeting agents.^7–9^

**Fig. 1:**
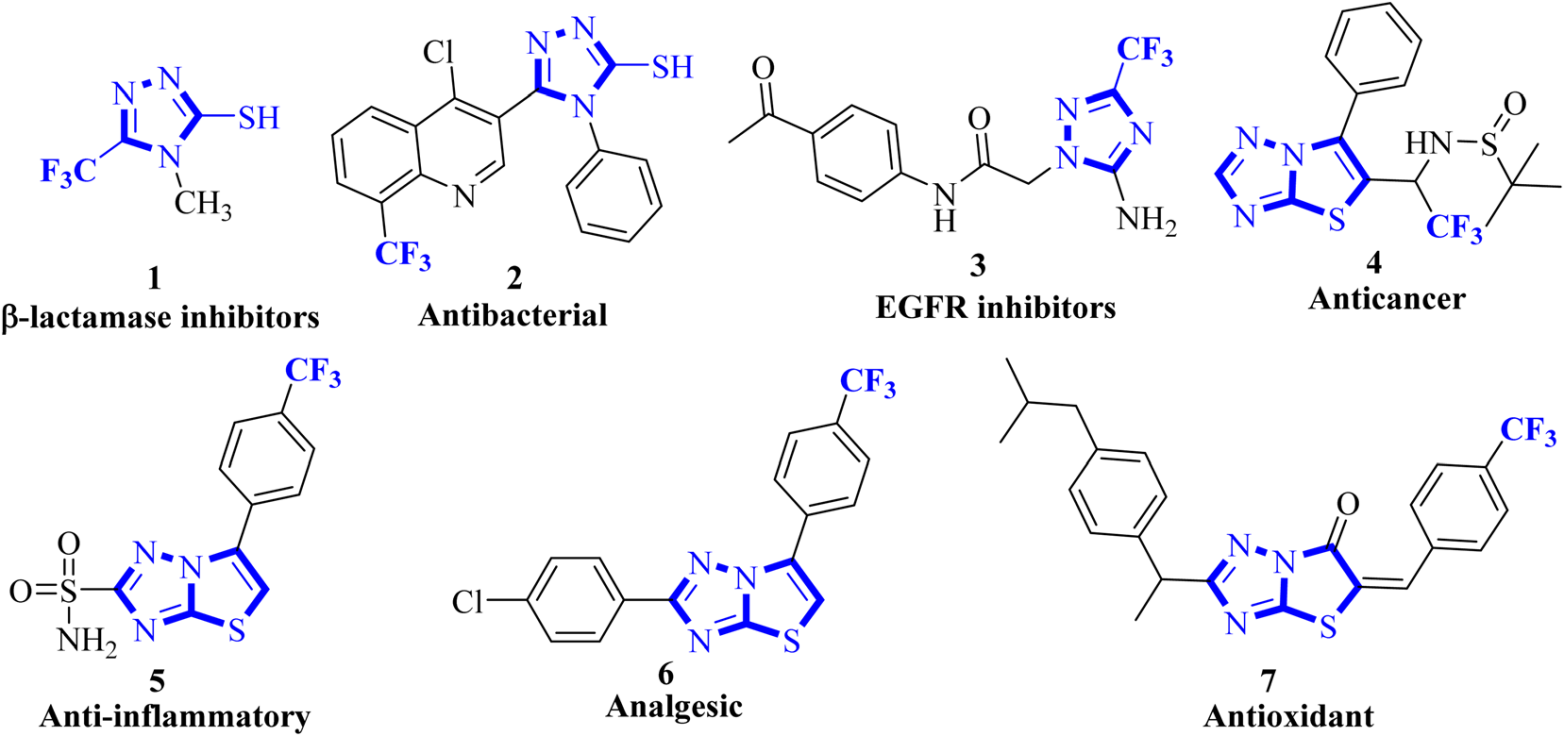
Bioactive fluorinated 1,2,4-triazole-based compounds.

1,2,4-Triazoles belong to an important class of heterocyclic compounds containing three nitrogen atoms in a five-membered aromatic ring. Triazoles are stable for metabolic degradation and show target specificity, as the three nitrogen atoms can act as hydrogen bond acceptors or donors at the active site of the biological receptors and can modulate their activity accordingly.^10^ Meanwhile, CF_3_ substituted 3-mercapto[1,2,4]triazole ring system and its fused derivative; thiazolo[3,2-*b*][1,2,4]triazoles represent an important group of bioactive compounds has captured remarkable attention from researchers owing to their wide array of applications, such as anticancer, anti-inflammatory, analgesic, antifungal, antibacterial, β-lactamase inhibitors, antiviral, and antioxidant agents.^11–14^ It is well documented that the integration of CF_3_ with 1,2,4-triazoles plays a substantial role in the drug development program and agrochemical industry.^15^ Some biologically representative examples of trifluoromethyl-linked mercapto[1,2,4]triazole-based analogs (1-7) are shown in Fig. 1.

Within the realm of pharmacology, DNA is a crucial biological target for the binding of small chemical entities, particularly those utilized in the treatment of cancer.^16–18^ The research indicated that small organic molecules interact with DNA in two distinct ways; either by intercalating between base pairs or by recognizing the grooves. The major groove offers more opportunities for hydrogen bonding, but its pocket for small molecules is broader and less deep than that of the minor groove. Hence, the minor groove is the preferred site for small ligands, which creates a more hydrophobic environment between the base pairs. In contrast, proteins and peptides tend to favour the major groove for binding.^19,20^ The minor groove of DNA plays a pivotal role in a variety of molecular interactions and target modifications, rendering it a significant point of interest. Drugs that bind to the minor groove of DNA have garnered considerable attention for their ability to combat tumor-related afflictions.^18,21^ In recent years, researchers have discovered compounds that bind to DNA and can alter its functions, offering the potential for new forms of chemotherapy. Through extensive biochemical studies, these compounds have been considered as having antibiotic, antiviral, or antitumor properties. Therefore, it is essential to investigate the dynamics of these interactions within the natural environment of living cells and the development of corresponding investigative tools.

Considering the unique properties of the CF_3_ group and the significant biological applications of 3-mercapto-1,2,4-triazole derivatives, it was deemed advantageous to incorporate the trifluoromethyl group into the triazole ring. Pertinent to the present research, the development of a general and convenient approach for the assembly of trifluoromethyl decorated 1,2,4-triazole-based derivatives is still highly desirable. In this context, we aimed to synthesize new trifluoromethylated isomers of thiazolo[3,2-*b*][1,2,4]triazole by reacting 3-mercapto[1,2,4]triazoles 8 with trifluoromethyl-β-diketones 9 in the presence of NBS. However, open-chain analogs were obtained which were investigated for DNA binding properties, and *ex vivo* studies were performed using various spectroscopic techniques.

## Results and discussion

**Scheme 1:**
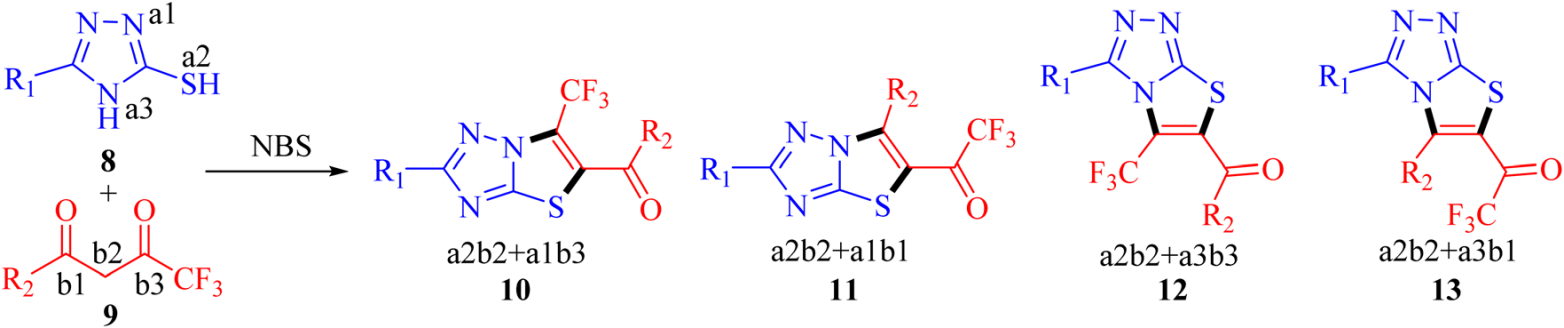
Proposed synthesis of fluorinated thiazolo[3,2-*b*][1,2,4]triazoles.

Recently, we have reported visible-light-mediated regioselective synthesis of thiazolo[3,2-*b*][1,2,4]triazoles by the reaction of 2-bromo-1,3-diketones, with 3-mercapto[1,2,4]triazoles under aqueous conditions in excellent yields.^22,23^ In the quest for the development of CF_3_-integrated novel therapeutic agents, we envisaged exploring the reaction of 3-mercapto[1,2,4]triazoles 8 with trifluoromethyl-β-diketones 9 in the presence of NBS to achieve the synthesis of trifluoromethylated thiazolo[3,2-*b*][1,2,4]triazoles. The reaction, in principle may afford four possible regioisomers; 5-aroyl-6-trifluoromethylthiazolo[3,2-*b*][1,2,4]triazoles 10, 6-aryl-5-trifluoroacetylthiazolo[3,2-*b*][1,2,4]triazoles 11, 6-aroyl-5-trifluoroacetylthiazolo[2,3-*c*][1,2,4]triazole 12 and 5-aryl-6-trifluoroacetylthiazolo[2,3-*c*][1,2,4]triazole 13, based on their fusion permutation, as illustrated in Scheme 1.

To elucidate our work hypothesis, 5-(4-methylphenyl)-3-mercapto[1,2,4]triazole 8a and 4,4,4-trifluoro-1-phenylbutane-1,3-dione 9a in the presence of NBS were selected as the model reaction partners to screen the reaction conditions. The reaction was monitored by TLC (ethyl acetate : petroleum ether, 30 : 70, *v/v* as eluent) at regular intervals. The first reaction was carried out under visible-light conditions; similar to those already successfully employed for the regioselective synthesis of non-fluorinated thiazolo[3,2-*b*]^1,2,4^ triazoles by our research group,^22,23^ however, the reaction could not be initiated even after irradiation of 5 h (run 1, Table 1). Thereafter, the reaction was explored in ethanol (EtOH) and a mixture of EtOH and H_2_O in different ratios under visible light, TLC indicated the partial consumption of reactants and resulted in low yields (runs 2-4, Table 1). Refluxing in EtOH pleasingly afforded the product in 71% yield after 2 h (run 5, Table 1), however, the use of PTSA as an additive did not improve the reaction outcomes (run 6, Table 1). Thereafter, the condensation reaction was screened in different solvents *viz*. DCM, THF, and DMF under refluxing conditions (run 7-9, Table 1), however, no significant results were observed in terms of yield or reaction time. Shifting the reaction conditions to a solvent-free environment caused a slowdown of the reaction rate and decreased the reaction yield (run 10-11, Table 1). Based on the screening results, the refluxing of substrates in EtOH was selected as the best condition for this transformation. In all the cases, we have notably observed the consistent formation of a single product with the same *R*_f_ values.

**Table 1:** Screening of the reaction conditions^a^

Run	Solvent	Visible light (27 W)/convention heat	Time	Yields^b^ (%)
1	H_2_O	Visible-light	5 h	nr
2	EtOH	Visible-light	5 h	25
3	EtOH : H_2_O (1 : 1)	Visible-light	5 h	20
4	EtOH : H_2_O (4 : 1)	Visible-light	5 h	30
**5**	**EtOH**	**Reflux**	**2 h**	**71**
6	EtOH/PTSA	Reflux	2 h	70
7	DCM	rt/reflux	5 h	nr
8	THF	Reflux	5 h	20
9	DMF	Reflux	5 h	15
10	Solvent-free	70–80 °C	5 h	15
11	Solvent-free/PTSA	70–80 °C	5 h	15

a Reaction of 8a (1.0 mmol), 9a (1.0 mmol), NBS (1.0 mmol) in different solvents was processed in the indicated reaction conditions.

b Isolated yield; nr: no reaction.

It is noteworthy to highlight here that inspired by Sherif *et al.* work on thiazolo[3,2-*b*][1,2,4]triazoles synthesis in acidified acetic acid (AcOH/H^+^),^24^ our study replicated the acidic conditions using 8a and 9a in presence of NBS. However, the outcomes closely mirrored our previous findings,^23^ revealing the exclusive formation of a single product identified as 6-phenyl-2-(4-methylphenyl)thiazolo[3,2-*b*][1,2,4]triazole, confirmed through comparison of its melting point and spectral data with literature values.^25^

**Scheme 2:**
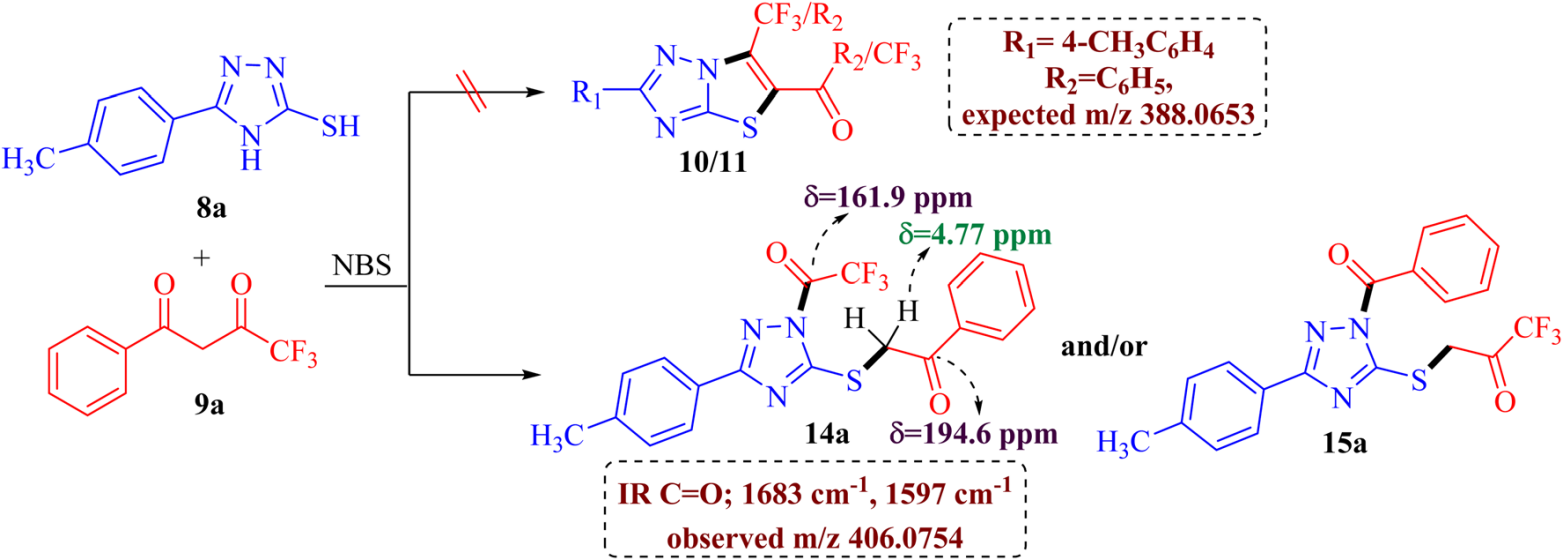
Unexpected product formation from the reaction of 8a and 9a.

The preliminary examination of the spectral data of the obtained product gestured towards some interesting reaction pathway of 5-(4-methylphenyl)-4*H*-3-mercapto[1,2,4]triazoles 8a with trifluoromethyl-β-diketone 9a to afford an unexpected product, which does not correlate with the data desired for the expected fluorinated thiazolotriazoles 10, 11, 12 or 13 (Scheme 2), as obtained in our earlier non-fluorinated thiazolotriazole analogs.^22,23^

The IR spectrum of the obtained product 14a/15a displayed an additional strong C=O absorption band at 1683 cm^−1^ along with the expected C=O absorption band at 1597 cm^−1^ indicating the presence of two carbonyl groups in the product. ^13^C NMR spectrum also depicted the presence of two carbonyl carbons at *δ* 194.6 ppm and 161.9 ppm along with the desired aromatic carbons, however, an additional peak for the methylene group at *δ* 39.2 ppm has been observed in the spectrum. Furthermore, the ^1^H NMR spectrum also depicted an additional singlet integrating for two protons in the aliphatic region (*δ* 4.77 ppm) indicating the formation of an unexpected open chain product from the unusual reaction of 8a and 9a. Additionally, the DEPT spectrum depicted the presence of five methines (CH), one methylene (CH_2_), and one methane (CH_3_) carbon. To be further certain about the structure; we have recorded the mass spectrum of the obtained product. The mass spectrum depicted an intense peak, *m*/*z* value of 406.0754 for [M+1] peak, which is notably higher than the expected value for the fluorinated thiazolotriazoles 10, 11, 12 or 13 value (388.0653). Thus, the preliminary examination of spectra indicated the formation of an open chain analog instead of cyclized thiazolotriazoles, which was identified as 1-trifluoroacetyl-3-(4-tolyl)-5-(2-oxo-2-phenylethylthio)-1,2,4-triazole 14a or 1-benzoyl-3-(4-tolyl)-5-(2-oxo-2-trifluoromethylethylthio)-1,2,4-triazole 15a by the combined analysis of spectral data including IR, 1D NMR and HRMS spectrum.

**Fig. 2:**
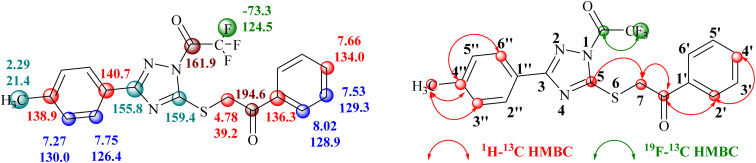
^1^H, ^13^C and ^19^F chemical shifts (in ppm) of compounds 14a and correlation illustration.

Conclusive evidence for the formation of 1-trifluoroacetyl-3-aryl-5-(2-oxo-2-arylethylthio)-1,2,4-triazole regioisomer 14 was obtained by collective results from the rigorous study of heteronuclear 2D NMR experiments of the compound 14a. The chemical shift values of protons and their corresponding carbons were assigned using (^1^H–^13^C) HSQC spectrum. Further, the (^1^H–^13^C) HMBC results showed cross-peaks of carbonyl carbon (*δ* 194.6 ppm) with 2′/6′-H proton (*δ* 8.02 ppm) of the phenyl ring indicating the presence of carbonyl carbon with aryl ring and the cross peak of carbonyl carbon (*δ* 194.6 ppm) with CH_2_ (*δ* 4.77 ppm) confirmed the presence of CH_2_–CO-Ar fragment, ruled out the possibility for the formation of regioisomer 15a. Similarly, the cross peak of C-5 (*δ* 159.4 ppm) of the triazole nucleus with CH_2_ (*δ* 4.77 ppm) indicated the presence of 2-oxo-2-arylethylthio group at position 5 of the triazole ring. Other correlations are seen from protons of CH_3_ (*δ* 2.29 ppm) with C-4′′ (*δ* 138.9 ppm), C-3′′/C-5′′ (*δ* 130.0 ppm). The (^19^F–^13^C) HMBC results displayed the correlation of fluorine (*δ* −73.4 ppm) with carbonyl carbon (*δ* 161.9 ppm). All expected correlations were validated in the COSY and NOESY spectrum, sufficient to support the substituents pattern around the triazole nucleus and all our data are consistent with structure 14a as the reaction product. The 2D NMR correlation results obtained for compounds 14a and their ^1^H, ^13^C, and ^19^F chemical shift values are presented in Fig. 2.

**Scheme 3:**
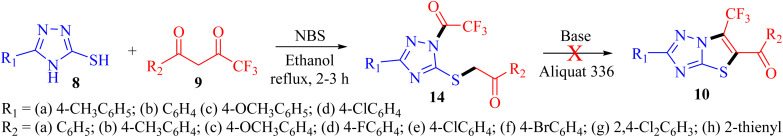
The general method for the synthesis of 14.

Following an in-depth structural analysis of the unexpected product, we made efforts to induce cyclization of the cleaved product 14 employing Aliquat 336 as a phase transfer catalyst and exploring diverse basic conditions (KOH, K_2_CO_3_, C_2_H_5_ONa, DABCO, and trimethylamine) to obtain the desired cyclized product 10. Unfortunately, none of these reaction experiments yielded the anticipated outcome (Scheme 3).

**Fig. 3:**
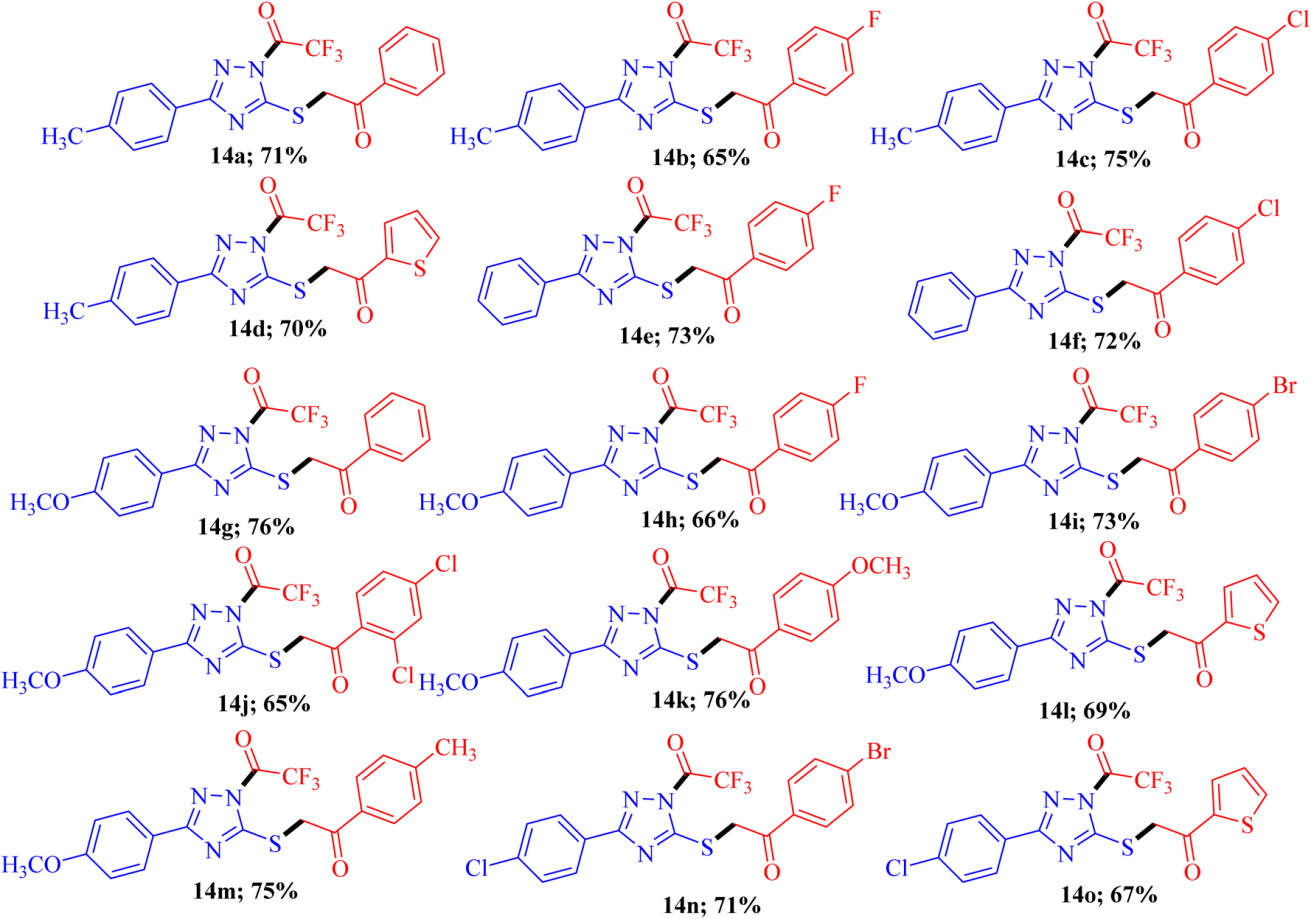
Substrate scope^*a*,*b*^. ^*a*^Reaction conditions: 3-mercapto[1,2,4]triazoles 8a-d (1.0 mmol), diketones 9a-h (1.0 mmol), and NBS (1.0 mmol) reflux in ethanol (10 ml) for 2-3 h; ^*b*^isolated yields.

To explore the biological potential of the newly synthesized compound, we conducted a comprehensive investigation into the substrate scope and limitations of this process. Variously substituted 3-mercapto[^1,2,4^]triazole and fluorinated 1,3-diketone compounds were employed, and the results are depicted in Fig. 3. The outcomes indicated that, in all cases, the reaction proceeded smoothly, yielding 14a–o as the final product. Although different substituent groups had a slight effect on the yields, the differences were minimal, and it was challenging to ascertain the specific impact of any individual substituent.

### Mechanism

**Scheme 4:**
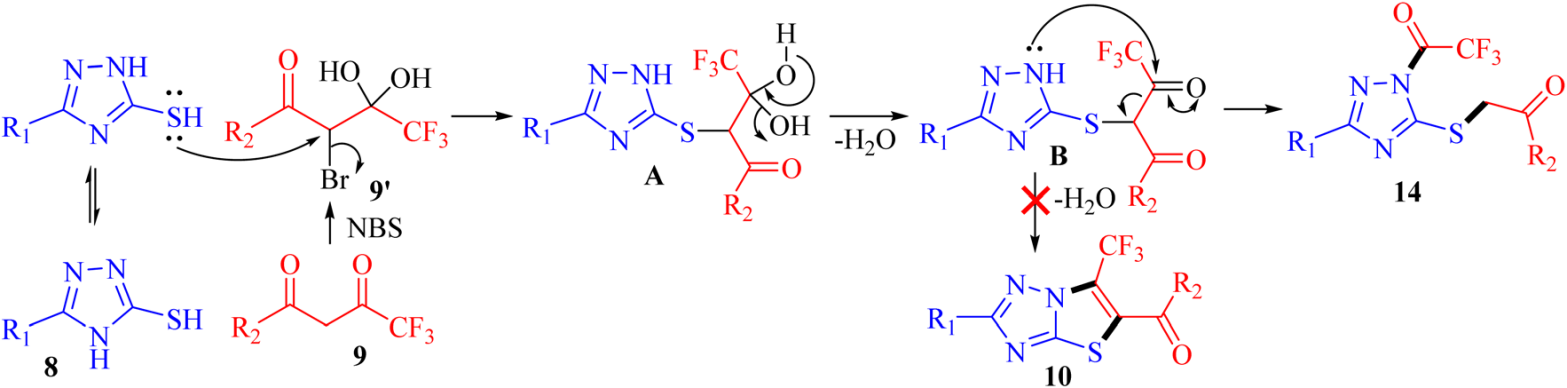
A possible mechanism for synthesis of 14.

The possible mechanism for the synthesis of 1-trifluoroacetyl-3-aryl-5-(2-oxo-2-arylethylthio)-1,2,4-triazoles 14a–o is outlined in Scheme 4. Based on the literature, the bromination of trifluoromethyl-β-diketones 9 with NBS resulted in 2-bromo-4,4,4-trifluoro-3,3-dihydroxybutan-1-ones 9′.^26,27^ The reaction is initiated by the nucleophilic displacement of bromine of 9′ by sulfur of 3-mercapto[1,2,4]triazole 8 to give an open chain intermediate A. Subsequently, dehydration from diol group to regenerate carbonyl provided the dicarbonyl intermediate B. Nucleophilic attack of amine nitrogen at more electrophilic carbonyl carbon adjacent to the CF_3_ group followed by C–C bond cleavage provided 1-trifluoroacetyl-3-aryl-5-(2-oxo-2-arylethylthio)-1,2,4-triazoles 14 as the final product instead of cyclization to yield 6-(trifluoromethyl)thiazolo[3,2-*b*][1,2,4]triazoles 10.

### DNA binding studies

#### Molecular docking studies

Computational chemistry plays a crucial role in identifying the formation of complexes between organic molecules and biological receptors.^28,29^ Therefore, we conducted an in-depth study to investigate the interaction between acyl-functionalized triazole derivatives and a double-strand DNA dodecamer d(CGCGAATTCGCG)_2_ (PDB ID: 1BNA) by applying molecular docking screenings to all synthesized compounds, denoted as 14a–o. Table 2 contains a summary of the calculated binding energy values, for the complexes between the receptor and ligand. Ligand 14m having 4-methoxyphenyl substitution on triazole and 4-methyl substituted diketone was found to be the most effective and strongest binder with the DNA dodecamer based on the docking conformations and lower DNA binding energy of ligand 14m. The best docking pose of compound 14m had a binding affinity of −9.0 kcal mol^−1^, visualized in BIOVIA Discovery Studio Visualizer.

**Table 2:** Molecular binding affinity of DNA-triazole complexes

Comp. No.	Binding affinity (kcal mol^−1^)
14a	−8.6
14b	−8.8
14c	−8.8
14d	−8.2
14e	−8.1
14f	−8.2
14g	−7.0
14h	−8.4
14i	−8.6
14j	−8.6
14k	−8.8
14l	−8.1
14m	−9.0
14n	−8.4
14o	−8.1
10a	−6.3
10b	−6.9
10c	−7.0
10e	−6.0
10g	−6.3
10h	−6.0
10j	−6.1
10k	−6.6
10l	−6.8
10o	−6.1
10n	−7.0
10p	−6.7
10m	−7.2
10q	−6.2
10r	−6.5

**Fig. 4:**
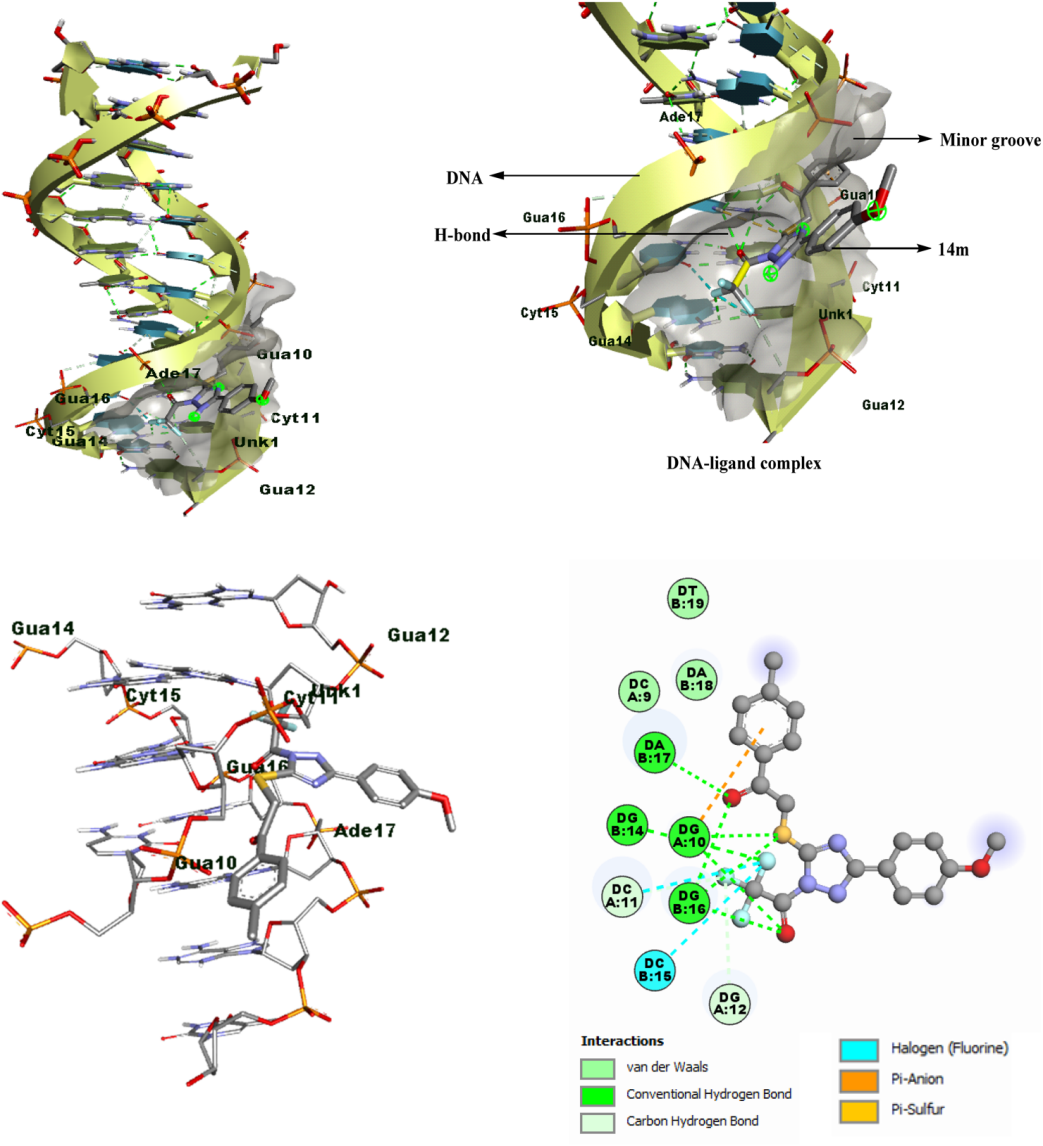
2D/3D binding poses of DNA-14m complex (PDB ID: 1BNA).

Through analysis of docking, it has been discovered that the triazole derivatives bind in the minor groove section of base pairs particularly in the guanine-rich region through various non-covalent interactions, such as hydrophobic interactions (π-alkyl, π-sulfur, *etc*), conventional hydrogen bonding, halogen interactions, and van der Waals forces. By examining the 2D plots of the best dock complex (DNA-14m), it was determined that both the carbonyl groups of the open-chain functionalized triazole derivative played a crucial role in conventional hydrogen bonding, with additional supportive interactions provided by the sulfur atom, and trifluoromethyl group (Fig. 4).

It is significant to mention that the corresponding cyclized derivatives 10 had a low docking score (−6.0 to −7.2 kcal mol^−1^), which may be attributed to the presence of only one carbonyl group, resulting in a decreased likelihood of H-bond formation. Additionally, the cyclized structure limits the flexibility of the molecule, thereby restricting its binding strength.

The docking analysis revealed that among all the compounds, 1-trifluoroacetyl-3-(4-methoxyphenyl)-5-((2-oxo-2-(4-tolyl)ethyl)thio)-1,2,4-triazole 14m had the best docking results. Therefore, it was chosen for further investigation through various spectral techniques to understand its interaction with calf thymus DNA and its mechanisms.

#### UV-visible studies

**Fig. 5:**
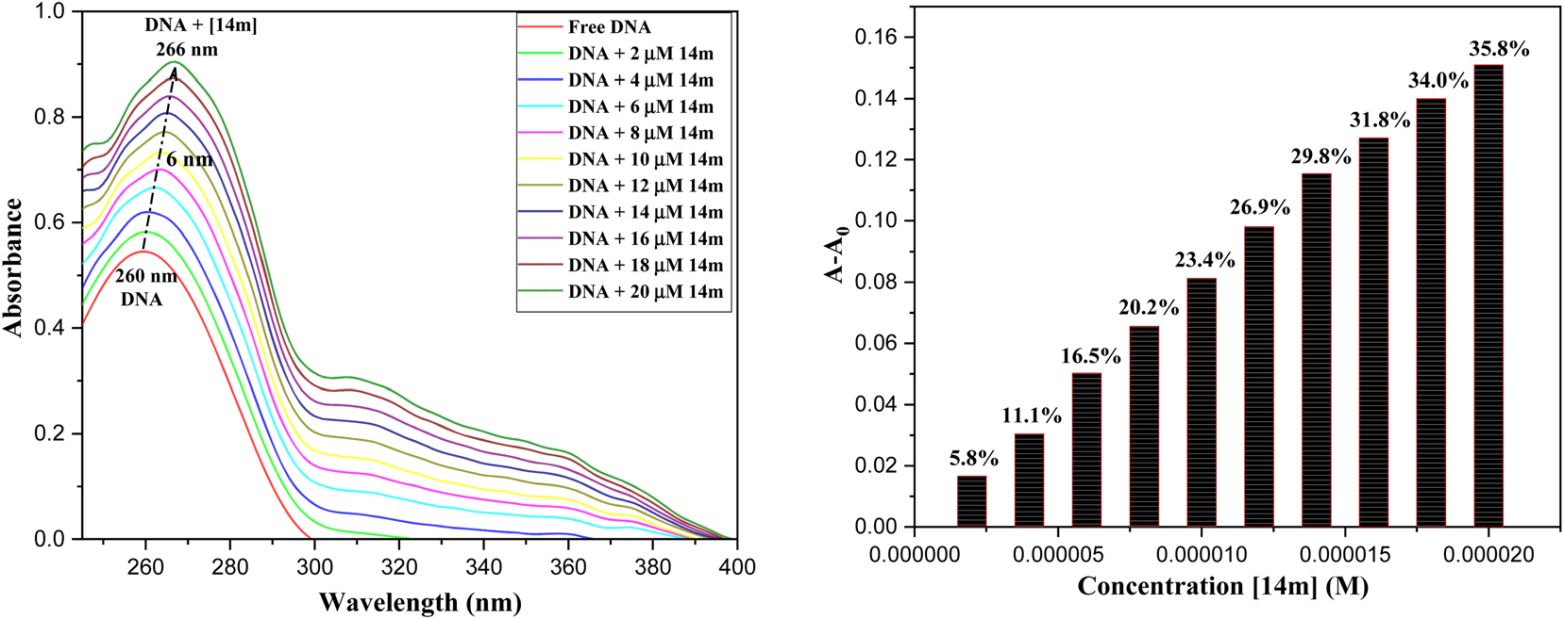
UV-visible absorption spectra of ctDNA titrating with 14m (0–20 μM).

Electronic absorption spectroscopy is a highly effective method for analyzing how organic molecules bind with DNA.^29^ Typically, changes in hyperchromism and hypochromism are the key spectral features of DNA's double-helix structure. Hyperchromism refers to the disruption of the secondary structure of DNA, while hypochromism suggests that the complex's binding mode with DNA is either an electrostatic effect or intercalation, which can stabilize the DNA duplex. The presence of a red shift indicates that the DNA duplex is being stabilized.^30^ To demonstrate the possibility of acyl-functionalized 1,2,4-triazoles binding with calf thymus (ct)DNA, spectroscopic titration of 14m with DNA has been performed for different ratios of [14m]/[DNA] while maintaining a constant DNA concentration (72 μM) in Tris-HCl buffer. Fig. 5 shows the UV spectra of DNA when exposed to different concentrations of 14m at 37 °C. The absorption spectra of ctDNA showed changes when titrated with triazole molecules, with an increase in intensity at the wavelength of 260 nm up to 35.8% with a 6 nm red shift (bathochromic shift) in the wavelength. This indicates that the interaction between the complexes and DNA occurs through the direct formation of a new complex with the double-helical ctDNA in the groove region. The increase in absorption intensity at 260 nm is due to the exposure of purine and pyrimidine bases in DNA caused by the binding of the complex to the DNA. This type of binding may have caused a slight change in the DNA's conformation.

After taking into consideration the aforementioned findings, we proceeded to delve deeper into how the DNA-triazole (14m) complex interacts. We aimed to gain a better understanding of the mode of interactions and binding strength. To this end, we utilized various methods such as fluorescence quenching, CD spectral, and viscosity analysis.

#### Fluorescence quenching studies

**Fig. 6:**
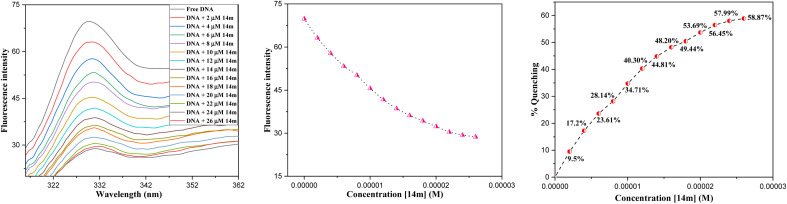
Fluorescence spectra of ctDNA titrating with 14m (0–28 μM) at 37 °C.

Fluorescence techniques have been highly effective in facilitating quantitative investigations of the equilibria and kinetics of drug-DNA interactions.^31,32^ The binding of organic species with biomolecules results in changes in fluorescence properties such as intensity or anisotropy. By utilizing equilibrium titration methods, these changes can be measured to accurately determine the stoichiometry, affinity, and thermodynamics of the binding interaction. Fig. 6 presents the emission spectra of ctDNA (72 μM) and its fluorescence titration with compound 14m (0–26 μM). The fluorescence spectra were recorded within the range of 300–500 nm at 37 °C, the peak excitation wavelength is at 290 nm, while the highest emission wavelength is at 330 nm. As the concentration of 14m increases, the fluorescence intensity of ctDNA rapidly decreases. The fluorescence is reduced by 58.87% when the DNA concentration rises to 26 μM.

**Fig. 7:**
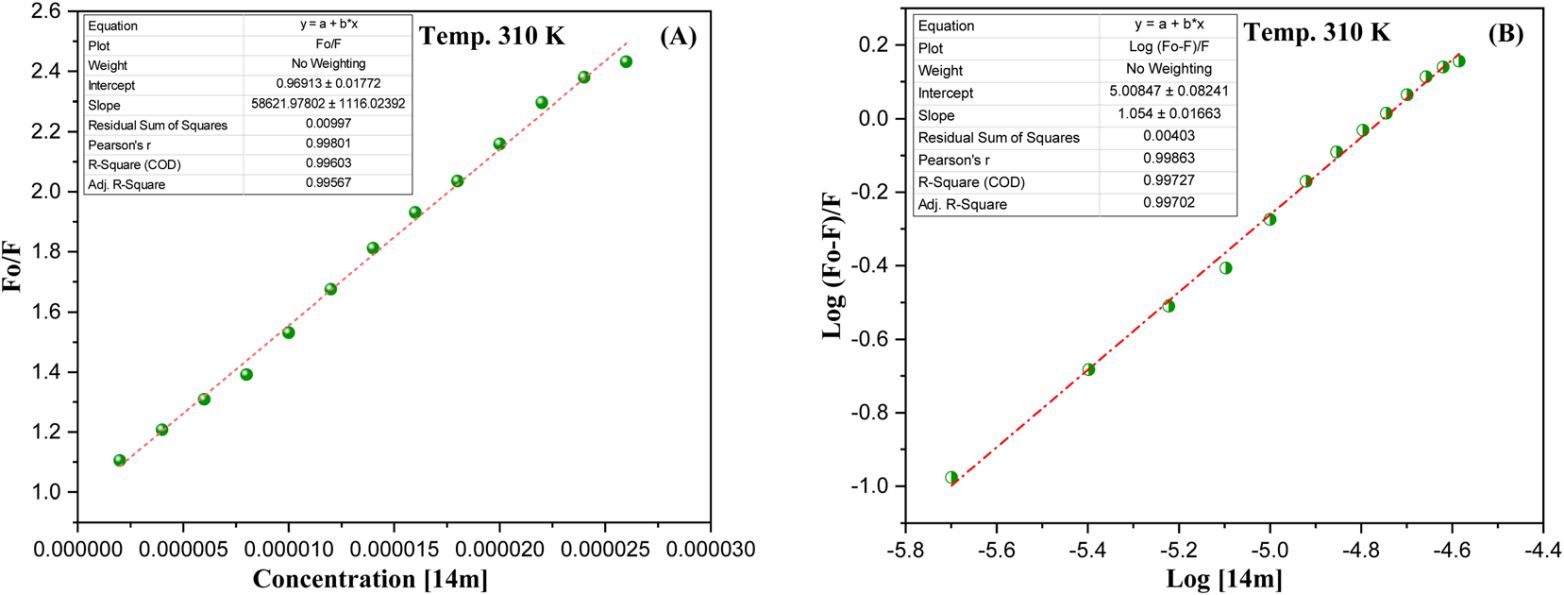
(A) Stern–Volmer; (B) Scatchard plots of DNA-14m complex.

The reduction in fluorescence intensity in the DNA spectrum is usually caused by quenching mechanisms such as static quenching, dynamic quenching, and mixed quenching. Static quenching occurs due to complex forms between biomolecule and quencher in the ground state, while dynamic quenching occurs due to collisions in the excited state. Through the utilization of Stern–Volmer constants (*K*_sv_) and quenching constants (*K*_q_), the efficacy of quenching in the spectrum can be comprehended. To this end, graphs were generated by plotting the ratio of fluorescence blank DNA and DNA+14m (*F*_0_/*F*) against increments of 14m, thus revealing the mechanism (Fig. 7A). The quenching constant was determined by assuming the average lifetime to be 10^−8^ seconds. The quenching constant (*K*_q_ = 5.86 ± 0.11 × 10^12^ M^−1^ s^−1^) was found to be higher than the scattering constant (10^10^ L mol^−1^ s^−1^), indicating that the quencher 14m forms a complex with DNA through static quenching.^33^

The value of the slope of the graphs determines the Stern–Volmer constant (*K*_sv_) (eqn (1)).1
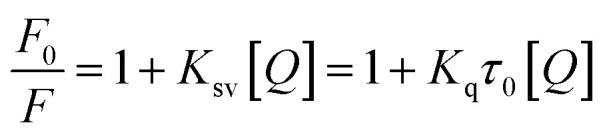


*F*_0_ represents the fluorescence intensity of pure DNA, however, *F* refers to the fluorescence intensity of DNA-14m complex (0–26 μM). [*Q*] signifies the molar concentration of 14m, *τ*_o_ denotes the average lifetime.

Furthermore, to understand how strongly DNA and 14m are bound together, we have calculated the intrinsic binding constant (*K*_b_) by creating Scatchard graphs (Fig. 7B). The binding constant *K*_b_ and number of binding sites *n* were calculated employing the Scatchard equation (eqn (2)) (Table 3).2
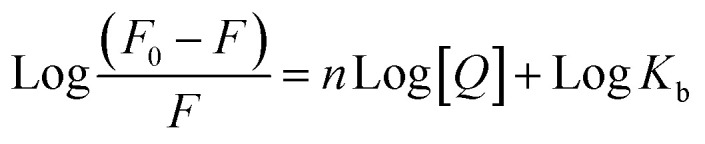


**Table 3:** Stern–Volmer constant (*K*_sv_), quenching constant (*K*_q_), binding constant (*K*_b_), the number of binding sites (*n*), and Gibbs free energy (Δ*G*°)

*K*_sv_ × 10^4^ (M^−1^)	*K*_q_ × 10^12^ (M^−1^ s^−1^)	Log K_*b*_	*K*_b_ × 10^5^ (M^−1^)	*n*	ΔG° (kJ mol^−1^)
(5.86 ± 0.11)	(5.86 ± 0.11)	5.00 ± 0.08	1.0	1.054 ± 0.01	−23.738

However, a change in Gibbs free energy (Δ*G*°) helps in the determination of the spontaneity of the binding procedures.

3 Δ*G*° = −RT ln *K*_b_

Δ*G*°, *K*_b_, and *R* refer to the Gibbs free energy, binding constant at temperature *T* and gas constant (8.314 J mol^−1^ K^−1^) respectively.

The intrinsic binding constant value has been determined to be within the range of 10^5^, signifying the robust and enduring binding between the triazole molecule and DNA through 1 : 1 binding stoichiometry. The results reveal the remarkable and effective bonding properties of the triazole compound with DNA, underscoring its potential as a valuable asset in diverse biological contexts. Additionally, the negative value for Gibbs free energy change Δ*G*° elegantly supports the feasibility and favorability for the formation of the DNA-14m complex.

**Fig. 8:**
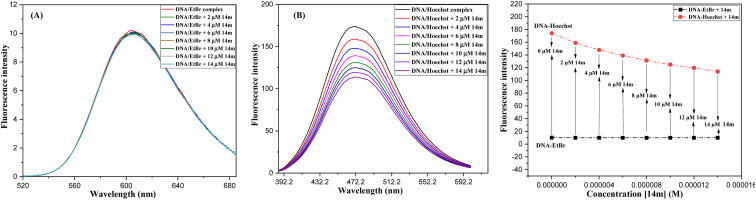
Competitive displacement assay of ctDNA-EtBr (A) and ctDNA-Hoechst 33 258 (B) in the presence of compound 14m.

The displacement assay is an important tool for identifying how DNA and a ligand molecule interact. Two well-known dyes, Ethidium bromide (intercalator) and Hoechst 33 258 (groove binder), are commonly used to determine whether a ligand molecule intercalates or binds to the grooves of DNA. The study tested the interaction of a triazole derivative (14m) with DNA using two different dyes (EtBr and Hoechst). The results showed that compound 14m did not displace the intercalating dye from the DNA-EtBr complex, indicating it does not interact with DNA through intercalation. However, there was a significant reduction in fluorescence intensity of the DNA-Hoechst complex with increasing concentration of compound 14m at 37 °C, suggesting it interacts with DNA through groove-binding modes (Fig. 8).

#### Circular dichroism (CD) spectroscopy

**Fig. 9:**
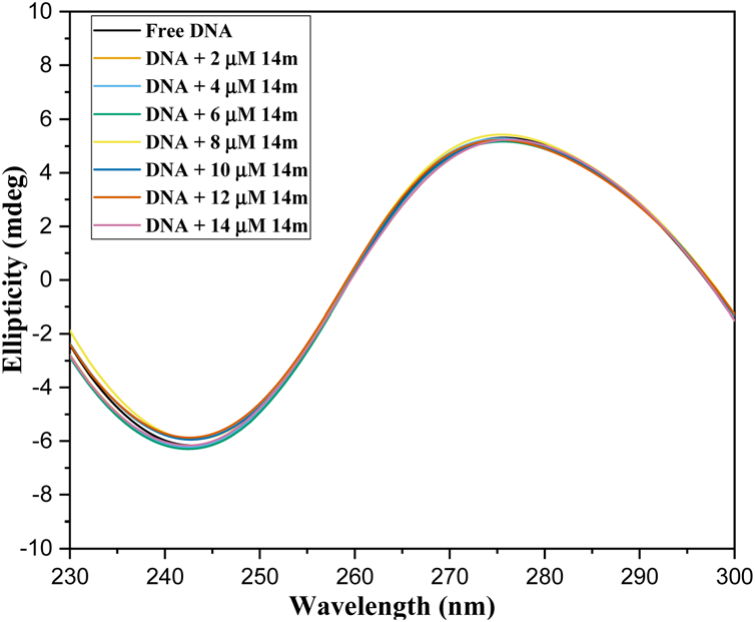
CD spectrum of ctDNA titrating with 14m.

CD spectroscopy proves to be valuable in identifying alterations in the structure of DNA as a result of drug-DNA interactions.^34,35^ The 275 nm positive band and 245 nm negative band, which correspond to base stacking and right-handed helicity respectively, are highly responsive to how small molecules interact with DNA. The modifications in the CD signals of DNA when exposed to drugs can usually be attributed to alterations in the DNA structure. Smaller molecules that bind to the grooves and have electrostatic interactions with DNA have little to no impact on the base-stacking and helicity bands. However, intercalation results in heightened intensities of both bands and stabilizes the DNA's right-handed B conformation, as demonstrated by methylene blue. The CD spectra of 14m and double-stranded DNA can offer valuable insights into their interactions. The CD spectrum of free DNA solution has a positive band at 275 nm and a negative band at 245 nm due to base stacking and helicity in the right-handed B form. Compound 14m has no CD spectrum alone, but has a titrated CD spectrum when interacting with DNA. The CD spectra of DNA did not display any significant change when titrated with 14m, indicating the groove-binding nature of the triazole derivative (Fig. 9). This data was consistent with results from UV-visible and fluorescence studies.

#### Viscosity measurements

**Fig. 10:**
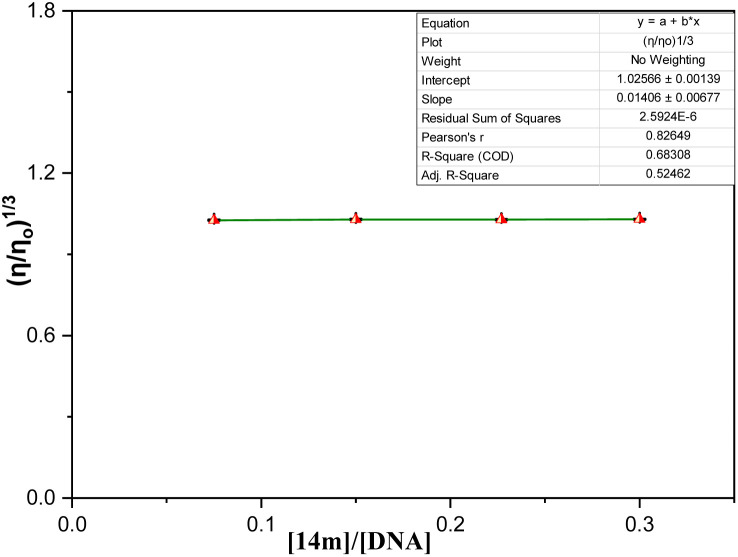
Relative viscosity of DNA by increasing the concentration of ligand 14m.

The measurement of viscosity in drug-DNA complexes is a reliable way to investigate their interaction.^36^ Intercalative binding, which occurs when a drug inserts itself between the base pairs of DNA, causes the DNA helix to lengthen and the viscosity to increase. However, if the drug interacts with DNA through electrostatic or groove binding, there is no significant change in viscosity. To examine the effect of 14m on CT-DNA, a graph of (*η*/*η*_o_)^1/3^*vs.* [14m]/[DNA] was plotted. The results showed that as 14m was added to the CT-DNA solution, the viscosity remained constant (Fig. 10). This indicated that 14m binds to DNA externally and did not intercalate into the DNA helix. The relative viscosity of the solution was determined using the relation below (eqn (4)):^19,37^4
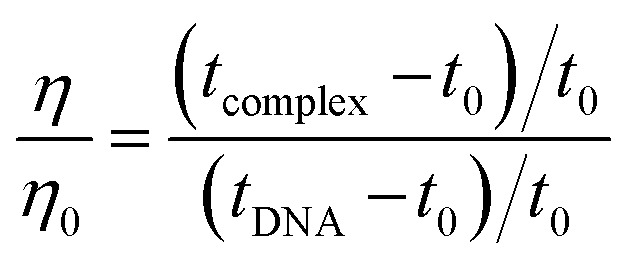
where *η*_0_ & *η* are the viscosities of DNA in the absence and presence of 14m respectively. *t*_DNA_, *t*_complex_, *t*_o_ is the average flow time of pure DNA, DNA-14m complex, and Tris-HCl buffer respectively.

## Conclusion

On the whole, a strategically novel one-step protocol for regioselective 1,3 *N*,*S*-difunctionalization of triazoles, which allows an expedient construction of CF_3_-containing architectures through an unprecedented reaction between 3-mercapto[1,2,4]triazoles and trifluoromethyl-β-diketones was disclosed. This methodology for the synthesis of various fluorinated triazoles has great potential for applications in related chemistry. *In silico* molecular docking analysis evidenced that trifluoromethylated triazole derivatives exhibited superior binding efficacy within the minor groove of the DNA double helix when compared to their cyclic counterparts. Spectroscopic results represented that binding of triazole derivatives to DNA resulted in significant changes in the structure and conformation of DNA in a concentration-dependent manner and acted as groove binder (*K*_b_ = 1 × 10^5^ M^−1^) *via* a static mode of quenching (*K*_q_ = 5.86 ± 0.11 × 10^12^ M^−1^ s^−1^). Moreover, circular dichroism and viscosity measurements supported the DNA groove binding nature of the triazole derivatives.

## Experimental

### Materials and methods

An electrical digital Melting Point Apparatus (MEPA) was used to examine melting points in open capillaries and were not corrected. Analytical TLC was performed using Merck Kieselgel 60 F254 silica gel plates and visualized under UV light (254 nm). IR spectra were recorded on Buck Scientific IR M-500 spectrophotometer in KBr pellets (*υ*_max_ in cm^−1^), ^1^H (400 Hz), and ^13^C NMR (100 Hz) spectra for the analytical purposes were recorded on a Bruker instrument, using CDCl_3_ and DMSO as a solvent and the chemical shifts are expressed in parts per million (ppm) and coupling constant *J* in Hz with TMS as internal standard. High-resolution mass spectra (HRMS) were measured in ESI^+^ mode at MRC, MNIT, Jaipur. 3-Mercapto-^1,2,4^ triazoles and 1,3-diketones were prepared as per the literature procedure,^23,38–41^ however, commercially available NBS (Avra Chemicals, India) were used without any purification.

### General procedure for the synthesis of 1-trifluoroacetyl-3-aryl-5-(2-oxo-2-arylethylthio)-1,2,4-triazoles 14a-o

Trifluoromethyl-β-diketones 9 (1.0 mmol) and NBS (0.178 g, 1.0 mmol) were homogenized thoroughly in a dry pestle mortar until a thick paste was formed and were subsequently added with an ethanolic solution of 5-aryl-3-mercapto[1,2,4]triazole 8 (1.0 mmol) in the round bottom flask. The reaction mixture was refluxed for 2–2.5 hours. The reaction progress was monitored with TLC using ethyl acetate-petroleum ether (30 : 70, *v/v*). On completion of the reaction, the solvent was evaporated at reduced pressure on the rotatory evaporator and the residue thus obtained was neutralized with a saturated solution of sodium bicarbonate and extracted with ethyl acetate. The obtained solid was recrystallized with ethanol and dried to afford pure 14 with 65–75% yields. The products were characterized by IR, ^1^H, ^13^C NMR, and HRMS spectrometry.

#### 1-Trifluoroacetyl-3-(4-tolyl)-5-((2-oxo-2-phenylethyl)thio)-1,2,4-triazole 14a

White solid; M.p. 143 °C; Yield 71%; IR (KBr, cm^−1^): 1683 (C=O), 1597 (C=O); ^1^H NMR (400 MHz, CDCl_3_) *δ* (ppm): 8.02–8.00 (m, 2H, 2′,6′-H), 7.75–7.73 (d, 2H, *J* = 8 Hz, 2′′,6′′-H), 7.65–7.62 (t, 1H, *J* = 8 Hz, 4′-H), 7.54–7.50 (t, 2H, *J* = 8 Hz, 3′,5′-H), 7.27–7.22 (m, 2H, 3′′,5′′-H), 4.77 (s, 2H, 7-CH_2_), 2.29 (s, 3H, 4′′-CH_3_); ^13^C NMR (100 MHz, CDCl_3_) *δ* (ppm): 194.6, 161.9, 159.4, 155.8, 140.7, 138.9, 136.3, 134.0, 130.1, 129.3, 128.9, 126.4, 124.5, 39.2, 21.4; ^19^F NMR (376 MHz, CDCl_3_) *δ* (ppm): −73.34; HRMS (ESI): 406.0754 [M + H]^+^.

#### 1-Trifluoroacetyl-3-(4-tolyl)-5-((2-oxo-2-(4-fluorophenyl)ethyl)thio)-1,2,4-triazole 14b

White solid; M.p. 152 °C; Yield 65%; IR (KBr, cm^−1^): 1680 (C=O), 1595 (C=O); ^1^H NMR (400 MHz, CDCl_3_) *δ* (ppm): 8.12–8.03 (m, 2H, 2′,6′-H), 7.74–7.72 (d, 2H, *J* = 8 Hz, 2′′,6′′-H), 7.38–7.33 (m, 2H, 3′,5′-H), 7.28–7.26 (d, 2H, *J* = 8 Hz, 3′′,5′′-H), 4.76 (s, 2H, 7-CH_2_), 2.30 (s, 3H, 4′′-CH_3_); ^13^C NMR (100 MHz, CDCl_3_) *δ* (ppm): 193.3, 164.4, 159.3, 155.8, 140.7, 133.0, 132.0–131.9 (d), 130.1, 129.7, 126.4, 126.1, 124.5, 116.4–116.2 (d), 39.1, 21.9; ^19^F NMR (376 MHz, CDCl_3_) *δ* (ppm): −73.32, −105.31; HRMS (ESI): 424.0664 [M + H]^+^.

#### 1-Trifluoroacetyl-3-(4-tolyl)-5-((2-oxo-2-(4-chlorophenyl)ethyl)thio)-1,2,4-triazole 14c

Yellow solid; M.p. 157 °C; Yield 75%; IR (KBr, cm^−1^): 1685 (C=O), 1600 (C=O); ^1^H NMR (400 MHz, CDCl_3_) *δ* (ppm): 8.05–8.01 (m, 2H, 2′,6′-H), 7.73–7.71 (m, 2H, 2′′,6′′-H), 7.63–7.57 (m, 2H, 3′,5′-H), 7.28–7.20 (m, 2H, 3′′,5′′-H), 4.75 (s, 2H, 7-CH2), 2.30 (s, 3H, 4′′-CH3); ^13^C NMR (100 MHz, CDCl_3_) *δ* (ppm): 193.8, 161.0, 159.3, 155.8, 140.7, 139.2, 135.0, 130.8, 130.1, 129.43, 129.42, 126.4, 124.4, 39.1, 21.5; ^19^F NMR (376 MHz, CDCl_3_) *δ* (ppm): −73.38; HRMS (ESI): 440.0368 [M + H]^+^; 442.0365 [M + H+2]^+^ (3 : 1).

#### 1-Trifluoroacetyl-3-(4-tolyl)-5-((2-oxo-2-(thienyl)ethyl)thio)-1,2,4-triazole 14d

Brown solid; M.p. 163 °C; Yield 70%; IR (KBr, cm^−1^): 1690 (C=O), 1600 (C=O); ^1^H NMR (400 MHz, CDCl_3_) *δ* (ppm): 8.12–8.10 (dd, 1H, *J* = 4 Hz, *J* = 1 Hz, 3′-H), 8.03–8.01 (dd, 1H, *J* = 4 Hz, *J* = 1 Hz, 5′-H), 7.74–7.72 (m, 2H, 2′′,6′′-H), 7.27–7.22 (m, 3H, 4′,3′′,5′′-H), 4.71 (s, 2H, 7-CH_2_), 2.29 (s, 3H, 4′′-CH_3_); ^13^C NMR (100 MHz, DMSO-d^6^) *δ* (ppm): 187.1, 161.6, 158.8, 155.3, 142.5, 140.2, 135.4, 134.2, 129.6, 128.9, 125.9, 123.9, 38.2, 21.0; ^19^F NMR (376 MHz, CDCl_3_) *δ* (ppm): −80.21; HRMS (ESI): 412.0320 [M + H]^+^.

#### 1-Trifluoroacetyl-3-phenyl)-5-((2-oxo-2-(4-fluorophenyl)ethyl)thio)-1,2,4-triazole 14e

Yellow solid; M.p. 172 °C; Yield 73%; IR (KBr, cm^−1^): 1700 (C=O), 1602 (C=O); ^1^H NMR (400 MHz, CDCl_3_) *δ* (ppm): 8.14–8.06 (m, 2H, 2′,6′-H), 7.85–7.83 (m, 2H, 2′′,6′′-H), 7.49–7.45 (m, 2H, 3′,5′-H), 7.40–7.33 (m, 3H, 3′′,4′′,5′′-H), 4.77 (s, 2H, 7-CH_2_); ^13^C NMR (100 MHz, CDCl_3_) *δ* (ppm): 193.3, 161.9, 159.5, 155.7, 133.0, 132.0–131.9 (d), 131.3, 130.9, 129.6, 129.1, 127.2, 126.5, 126.2, 124.4, 116.4–116.2 (d), 39.2; ^19^F NMR (376 MHz, CDCl_3_) *δ* (ppm): −72.88, −104.98; HRMS (ESI): 410.0505 [M + H]^+^.

#### 1-Trifluoroacetyl-3-phenyl-5-((2-oxo-2-(4-chlorophenyl)ethyl)thio)-1,2,4-triazole 14f

Yellow solid; M.p. 173 °C; Yield 72%; IR (KBr, cm^−1^): 1695 (C=O), 1602 (C=O); ^1^H NMR (400 MHz, CDCl_3_) *δ* (ppm): 8.05–8.01 (m, 2H, 2′,6′-H), 7.85–7.82 (m, 2H, 2′′,6′′-H), 7.62–7.58 (m, 2H, 3′,5′-H), 7.49–7.42 (m, 2H, 3′′,5′′-H), 7.39–7.36 (m, 1H, 4′′-H), 4.77 (s, 2H, 7-CH_2_); ^13^C NMR (100 MHz, DMSO-d_6_) *δ* (ppm): 193.7, 161.8, 159.4, 155.6, 151.8, 138.8, 134.9, 130.7, 129.5, 129.3, 129.1, 127.0, 126.4, 126.1, 39.1; ^19^F NMR (376 MHz, CDCl_3_) *δ* (ppm): −74.21; HRMS (ESI): 426.0200 [M + H]^+^; 428.0206 [M + H+2]^+^ (3 : 1).

#### 1-Trifluoroacetyl-3-(4-methoxyphenyl)-5-((2-oxo-2-phenylethyl)thio)-1,2,4-triazole 14g

White solid; M.p. 175 °C; Yield 76%; IR (KBr, cm^−1^): 1683 (C=O), 1604 (C=O); ^1^H NMR (400 MHz, CDCl_3_) *δ* (ppm): 8.03–8.01 (m, 2H, 2′′,6′′-H), 7.86–7.84 (d, 2H, 2′,6′-H, ^*3*^*J* = 8.8), 7.62–7.60 (m, 1H, 4′-H), 7.50–7.46 (m, 2H, 3′′,5′′-H), 6.93–6.91 (d, 2H, 3′,5′-H, ^*3*^*J* = 8.8), 4.66 (s, 2H, 7-CH_2_), 3.83 (s, 3H, OCH_3_); ^13^C NMR (100 MHz, DMSO-d_6_) *δ* (ppm): 194.6, 161.3, 159.2, 155.6, 136.2, 134.0, 129.3, 128.9, 128.1, 127.6, 119.7, 115.0, 114.5, 55.8, 38.9; ^19^F NMR (376 MHz, CDCl_3_) *δ* (ppm): −72.54; HRMS (ESI): 422.0712 [M + H]^+^.

#### 1-Trifluoroacetyl-3-(4-methoxyphenyl)-5-((2-oxo-2-(4-fluorophenyl)ethyl)thio)-1,2,4-triazole 14h

Yellowish solid; M.p. 164 °C; Yield 66%; IR (KBr, cm^−1^): 1682 (C=O), 1602 (C=O); ^1^H NMR (400 MHz, DMSO-d_6_) *δ* (ppm): 7.99–7.97 (m, 2H, 2′,6′-H), 7.82–7.80 (m, 2H, 2′′,6′′-H), 7.79–7.77 (m, 2H, 3′,5′-H), 7.06–6.98 (m, 2H, 3′′,5′′-H), 4.78 (s, 2H, 7-CH_2_), 3.81 (s, 3H, OCH_3_); ^13^C NMR (100 MHz, DMSO-d_6_) *δ* (ppm): 193.5, 160.8, 158.6, 155.1, 134.8, 131.8, 130.4, 127.6, 127.1, 119.2, 114.4, 114.0, 55.3, 38.6; ^19^F NMR (376 MHz, DMSO-d_6_) *δ* (ppm): −78.13, −105.43; HRMS (ESI): 440.0618 [M + H]^+^.

#### 1-Trifluoroacetyl-3-(4-methoxyphenyl)-5-((2-oxo-2-(4-bromophenyl)ethyl)thio)-1,2,4-triazole 14i

Brownish solid; M.p. 181 °C; Yield 73%; IR (KBr, cm^−1^): 1688 (C=O), 1605 (C=O); ^1^H NMR (400 MHz, DMSO-d_6_) *δ* (ppm): 7.99–7.97 (m, 2H, 2′′,6′′-H), 7.86–7.79 (m, 4H, 2′,3′,5′,6′-H), 7.07–7.05 (m, 2H, 3′′,5′′-H), 4.78 (s, 2H, 7-CH_2_), 3.81 (s, 3H, OCH_3_); ^13^C NMR (100 MHz, DMSO-d_6_) *δ* (ppm): 193.5, 160.8, 158.5, 155.1, 134.8, 131.8, 130.3, 127.6, 127.2, 119.2, 114.4, 113.9, 55.3, 38.5; ^19^F NMR (376 MHz, DMSO-d_6_) *δ* (ppm): −73.32; HRMS (ESI): 500.9815 [M + H]^+^.

#### 1-Trifluoroacetyl-3-(4-methoxyphenyl)-5-((2-oxo-2-(2,4-dichlorophenyl)ethyl)thio)-1,2,4-triazole 14j

White solid; M.p. 186 °C; Yield 65%; IR (KBr, cm^−1^): 1684 (C=O), 1600 (C=O); ^1^H NMR (400 MHz, DMSO-d_6_) *δ* (ppm): 8.18–8.11 (m, 1H, 6′-H), 8.07–8.06 (m, 1H, 3′-H), 7.84–7.77 (m, 2H, 2′′,6′′-H), 7.30–7.28 (m, 1H, 5′), 7.06–7.04 (m, 2H, 3′′,5′′-H), 4.72 (s, 2H, 7-CH_2_), 3.80 (s, 3H, OCH_3_); ^13^C NMR (100 MHz, DMSO-d_6_) *δ* (ppm): 187.3, 161.0, 158.8, 157.2, 155.4, 142.5, 135.5, 6134.6, 134.3, 129.0, 127.8, 127.3, 119.2, 114.6, 112.0, 55.4, 38.3; ^19^F NMR (376 MHz, DMSO-d_6_) *δ* (ppm): −74.41; HRMS (ESI): 489.9930 [M + H]^+^.

#### 1-Trifluoroacetyl-3-(4-methoxyphenyl)-5-((2-oxo-2-(4-methoxyphenyl)ethyl)thio)-1,2,4-triazole 14k

White solid; M.p. 173 °C; Yield 76%; IR (KBr, cm^−1^): 1681 (C=O), 1596 (C=O); ^1^H NMR (400 MHz, CDCl_3_) *δ* (ppm): 7.99–7.97 (m, 2H, 2′′,6′′-H), 7.88–7.86 (m, 2H, 2′,6′-H), 6.93–6.89 (m, 4H, 3′′,5′′,3′,5′-H), 4.60 (s, 2H, 7-CH_2_), 3.86 (s, 3H, OCH_3_), 3.81 (s, 3H, OCH_3_); ^13^C NMR (100 MHz, CDCl_3_) *δ* (ppm): 193.0, 164.2, 161.0, 131.1, 128.1, 127.9, 120.8, 114.1, 113.9, 55.5, 55.3, 39.3; ^19^F NMR (376 MHz, CDCl_3_) *δ* (ppm): −73.22; HRMS (ESI): 452.0816 [M + H]^+^.

#### 1-Trifluoroacetyl-3-(4-methoxyphenyl)-5-((2-oxo-2-thienylethyl)thio)-1,2,4-triazole 14l

Greyish solid; M.p. 161 °C; Yield 69%; IR (KBr, cm^−1^): 1688 (C=O), 1602 (C=O); ^1^H NMR (400 MHz, DMSO-d_6_) *δ* (ppm): 8.16–8.14 (m, 1H, 3′-H), 7.92–7.90 (m, 1H, 5′-H), 7.83–7.81 (m, 2H, 2′′,6′′-H), 7.45–7.37 (m, 1H, 4′), 7.07–7.05 (m, 2H, 3′′,5′′-H), 4.79 (s, 2H, 7-CH_2_), 3.82 (s, 3H, OCH_3_); ^13^C NMR (100 MHz, DMSO-d_6_) *δ* (ppm): 192.7, 166.4, 163.9, 160.7, 158.6, 155.1, 132.4, 131.5, 131.4, 127.6, 119.2, 115.9, 115.7, 114.4, 55.3, 38.6; ^19^F NMR (376 MHz, DMSO-d_6_) *δ* (ppm): −72.11; HRMS (ESI): 428.0277 [M + H]^+^.

#### 1-Trifluoroacetyl-3-(4-methoxyphenyl)-5-((2-oxo-2-(4-tolyl)ethyl)thio)-1,2,4-triazole 14m

White solid; M.p. 169 °C; Yield 75%; IR (KBr, cm^−1^): 1685 (C=O), 1598 (C=O); ^1^H NMR (400 MHz, CDCl_3_) *δ* (ppm): 7.94–7.92 (m, 2H, 2′′,6′′-H), 7.89–7.87 (m, 2H, 3′,5′-H), 7.30–7.28 (m, 2H, 3′′,5′′-H), 7.54–7.45 (m, 2H, 2′,6′-H), 4.61 (s, 2H, 7-CH_2_), 3.84 (s, 3H, OCH_3_); ^13^C NMR (100 MHz, DMSO-d_6_) *δ* (ppm): 194.1, 161.3, 159.3, 155.6, 144.5, 133.7, 129.8, 129.0, 128.1, 127.6, 119.7, 115.0, 114.5, 55.8, 39.1, 21.7; ^19^F NMR (376 MHz, CDCl_3_) *δ* (ppm): −79.18; HRMS (ESI): 436.0866 [M + H]^+^.

#### 1-Trifluoroacetyl-3-(4-chlorophenyl)-5-((2-oxo-2-(4-bromophenyl)ethyl)thio)-1,2,4-triazole 14n

Brownish solid; M.p. 183 °C; Yield 71%; IR (KBr, cm^−1^): 1689 (C=O), 1606 (C=O); ^1^H NMR (400 MHz, DMSO-d_6_) *δ* (ppm): 8.08–8.06 (m, 2H, 2′′,6′′-H), 7.90–7.88 (m, 2H, 2′,6′-H), 7.84–7.74 (m, 4H, 3′,5′,3′′,5′′-H), 4.81 (s, 2H, 7-CH_2_); ^13^C NMR (100 MHz, DMSO-d_6_) *δ* (ppm): 188.0, 165.9, 146.0, 135.4, 132.9, 132.7, 131.9, 131.5, 130.0, 129.6, 129.4, 128.5, 128.4, 128.0, 39.2; ^19^F NMR (376 MHz, DMSO-d_6_) *δ* (ppm): −72.88; HRMS (ESI): 503.9320 [M + H]^+^.

#### 1-Trifluoroacetyl-3-(4-chlorophenyl)-5-((2-oxo-2-thienylethyl)thio)-1,2,4-triazole 14o

Greyish solid; M.p. 171 °C; Yield 67%; IR (KBr, cm^−1^): 1684 (C=O), 1604 (C=O); ^1^H NMR (400 MHz, DMSO-d_6_) *δ* (ppm): 8.12–8.10 (m, 1H, 6′-H), 8.04–8.02 (m, 1H, 3′-H), 7.86–7.83 (m, 2H, 2′′,6′′-H), 7.52–7.50 (m, 2H, 3′′,5′′-H), 7.27–7.24 (m, 2H, 5′-H), 4.74 (s, 2H, 7-CH_2_); ^13^C NMR (100 MHz, DMSO-d_6_) *δ* (ppm): 187.0, 159.3, 154.2, 142.4, 135.8, 135.4, 134.5, 134.2, 129.2, 128.9, 128.8, 127.7, 127.3, 125.5, 38.7; ^19^F NMR (376 MHz, DMSO-d_6_) *δ* (ppm): −73.34; HRMS (ESI): 431.9779 [M + H]^+^.

### DNA binding studies

#### Molecular docking study

The synthesized triazole derivatives were drawn using ChemDraw Professional 15.0 and underwent energy minimization *via* Chem3D 15.0 software. All ligands were processed and saved in the necessary format for molecular docking analyses. Following the initial phase, we acquired the 3D DNA file (B form) from the esteemed Protein Data Bank (RCSB) (http://www.rcsb.org/pdb) under the PDB ID 1BNA (DNA (5′-(CGCGAATTCGCG)-3′)). The file was subject to energy minimization for molecular docking, after which water molecules were removed and polar hydrogens were added. The docking examinations were performed through Auto Dock Vina (MGL tools), with the docked poses being meticulously analyzed using BIOVIA Discovery Studio Visualizer v21.1.0.20298.

#### Spectroscopic analysis

The ctDNA, purchased from Sigma-Aldrich, India, was of high purity as determined by absorption spectroscopy with an attenuance ratio *A*_260_/*A*_280_ between 1.8 and 1.9. Therefore, it was used without further purification. The DNA concentration was measured using spectrophotometry at a physiological pH of 7.4 and an extinction coefficient value of 6600 L mol^−1^ cm^−1^ at 260 nm wavelength.

Absorbance values were recorded on a double beam Thermo Scientific's Evolution 300 spectrophotometer and fluorescence measurements were performed on a Hitachi F-4700 quantum north-west 5J20700 fortified with a xenon lamp, using a quartz cuvette of path length 1 cm. The CD measurements of the DNA-14m complex (Far-UV, 200–250 nm) were recorded on a J-815 spectrophotometer (JASCO, Japan) at room temperature using a quartz cuvette with a path length of 10 mm.

#### Preparation of stock solution

A solution containing 14m at a concentration of 1 mM was made in DMSO. A uniform mixture of ctDNA was created by stirring it in Tris-HCl buffer using a vortex. The DNA concentration was measured at 72 μM using Beer–Lambert Law with a molar extinction coefficient of 6600 L mol^−1^ cm^−1^ for a single DNA strand.

## Author contributions

R. A. and S. K.; conceptualization, supervision, reviewing and editing. P. K. and M. H.; methodology, investigation, computational calculations, writing and original draft preparation.

## Conflicts of interest

The authors declare no competing interest.

## Supplementary Material

RA-014-D4RA00083H-s001
