# Supplementary material for: Serendipitous N,S-difunctionalization of triazoles with trifluoromethyl-β-diketones: access to regioisomeric 1-trifluoroacetyl-3-aryl-5-(2-oxo-2-arylethylthio)-1,2,4-triazoles as DNA-groove binders
Source: RSC Adv. 2024 Feb 23;14(10):6738–51. doi: 10.1039/d4ra00083h (PMC10884789; doi:10.1039/d4ra00083h)
Supplement: RA-014-D4RA00083H-s001 [file RA-014-D4RA00083H-s001.pdf]

Electronic Supplementary Material (ESI) for RSC Advances.
This journal is © The Royal Society of Chemistry 2024

Serendipitous *N,S*-Difunctionalization of Triazoles with Trifluoromethyl-β-diketones: Access to Regioisomeric 1-Trifluoroacetyl-3-aryl-5-(2-oxo-2-arylethylthio)-1,2,4-triazoles as DNA-Groove Binders

Ranjana Aggarwal,[a,b]* Prince Kumar,[a] Mona Hooda,[c] Suresh Kumar,[a]

[a]Department of Chemistry, Kurukshetra University, Kurukshetra-136119, Haryana, India

[b]Council of Scientific and Industrial Research-National Institute of Science Communication and Policy Research, New Delhi 110012, India.

[c]Department of Chemistry, Gurugram University, Gurugram-122003, Haryana, India.

*Corresponding author:

Prof. Ranjana Aggarwal, CSIR-National Institute of Science Communication and Policy Research, New Delhi, India. Tel: +91-9896740740

E-mails: *ranjana67in@yahoo.com*, *ranjanaaggarwal67@gmail.com*

SUPPLEMENTARY DATA

1-Trifluoroacetyl-3-(4-tolyl)-5-((2-oxo-2-phenylethyl)thio)-1,2,4-triazole 14a

^{1}H of **14a**

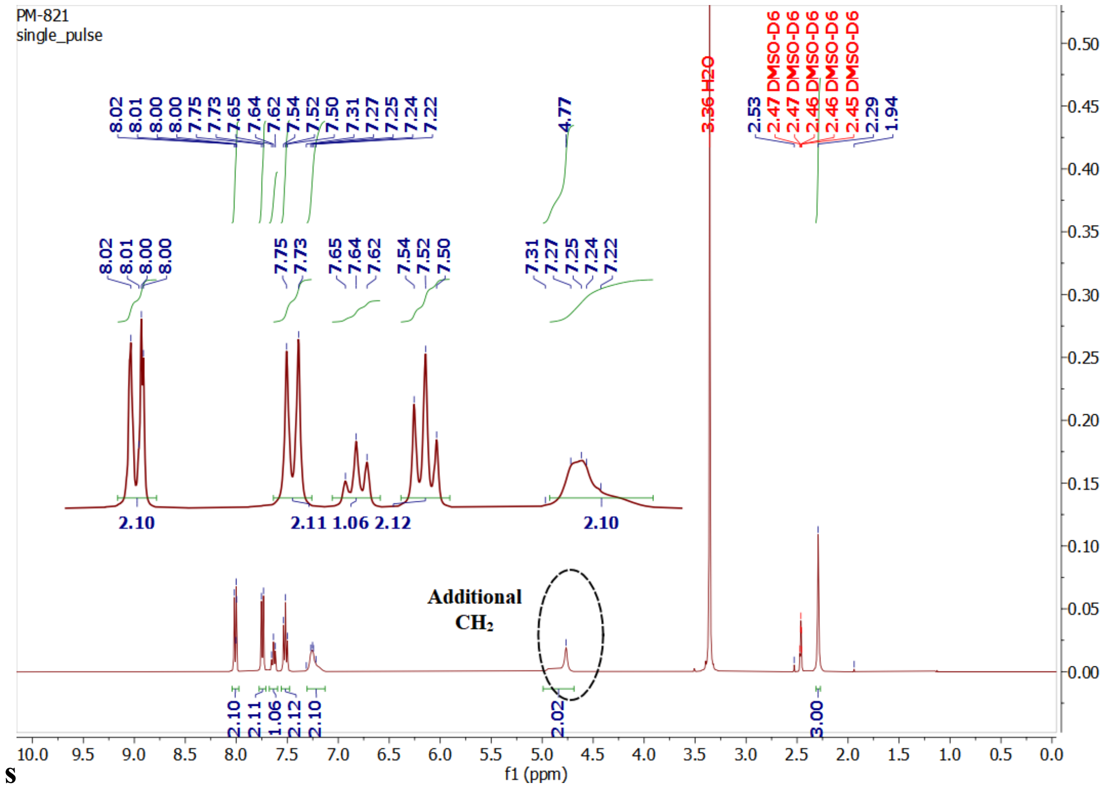

^{13}C of **14a**

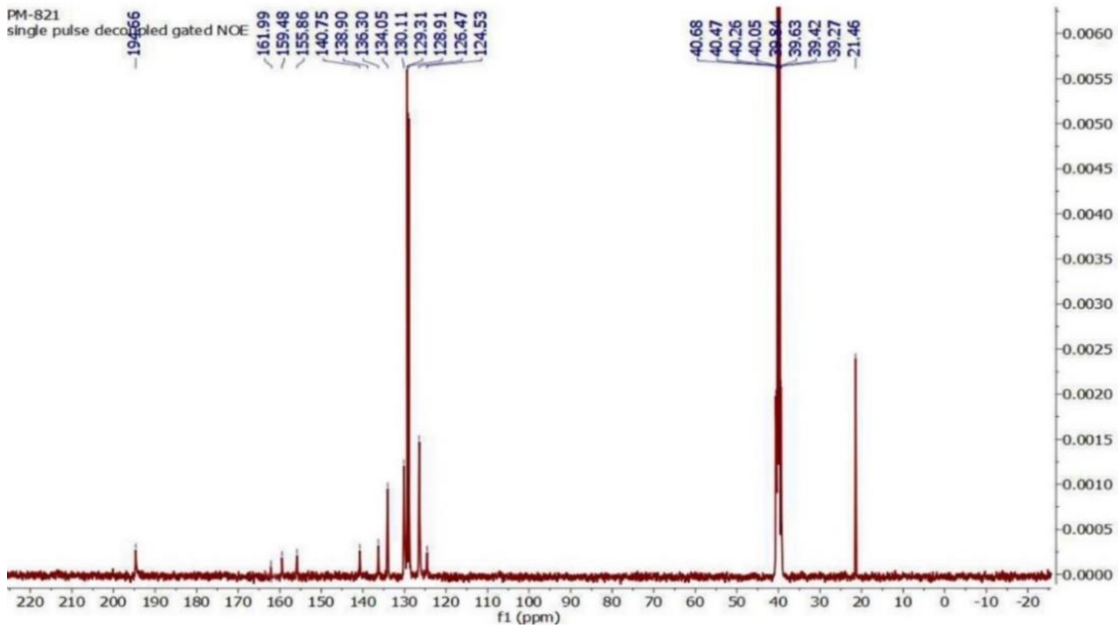

^{19}F of **14a**

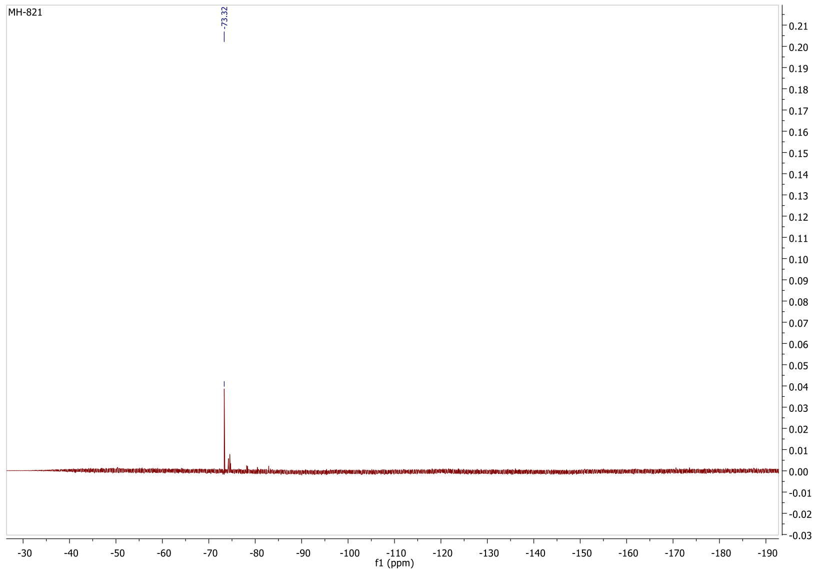

DEPT spectrum of **14a**

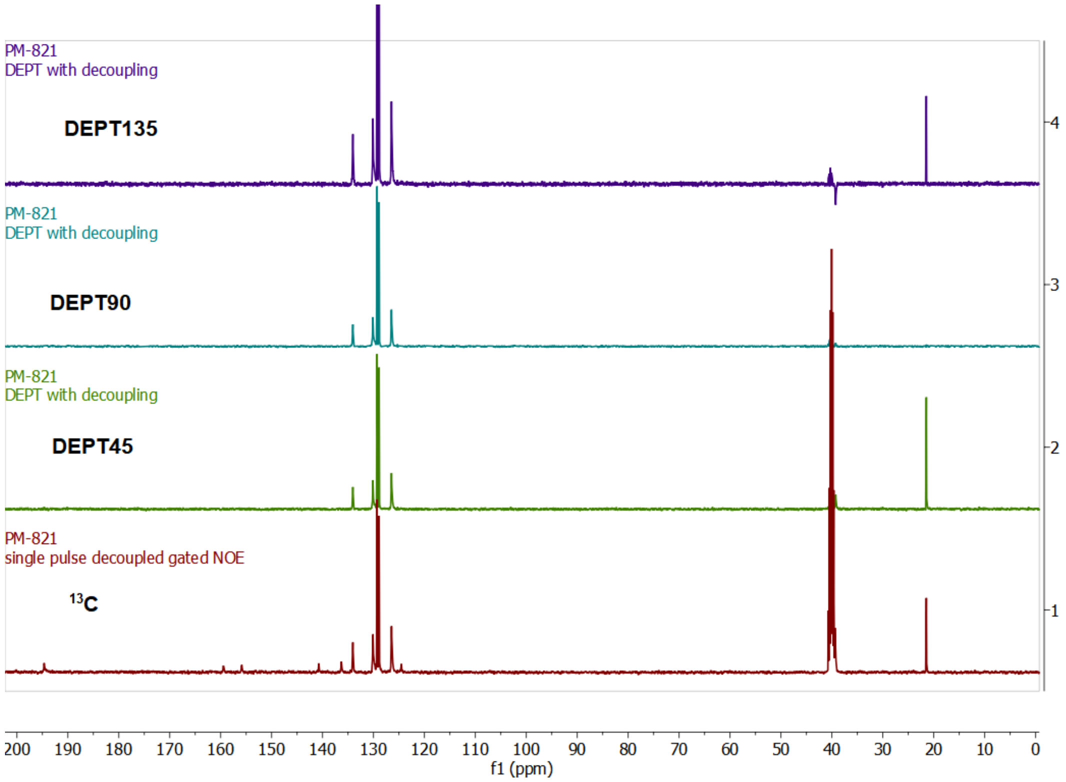

HRMS spectrum of compound **14a**

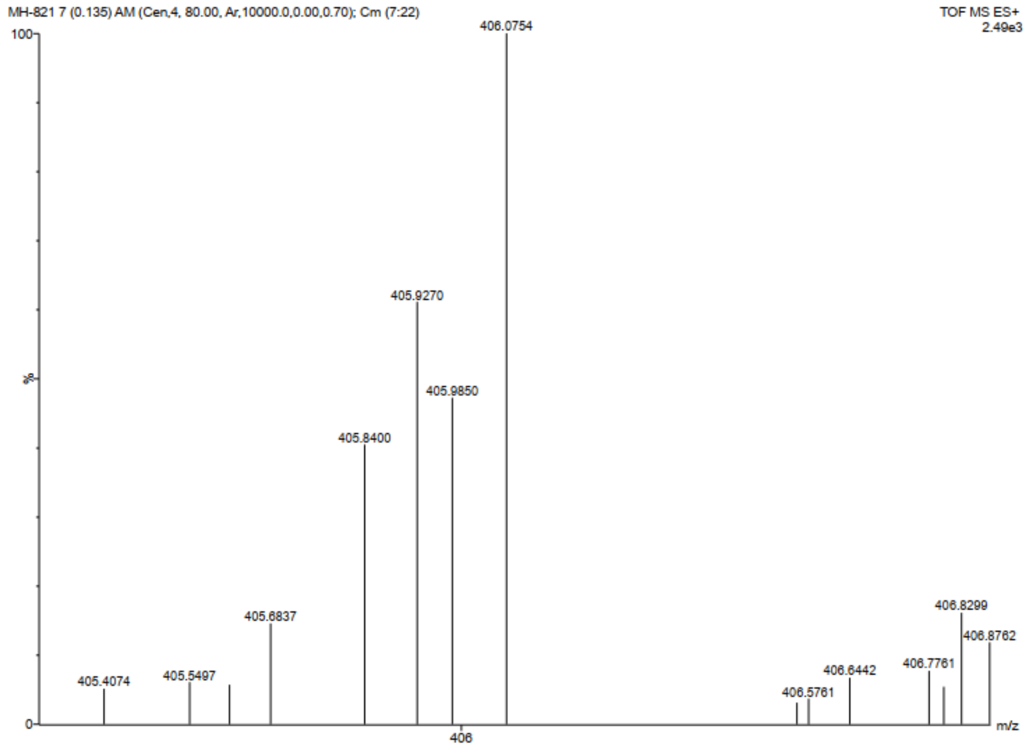

(^{1}H-^{13}C) HSQC spectrum of compound **14a**

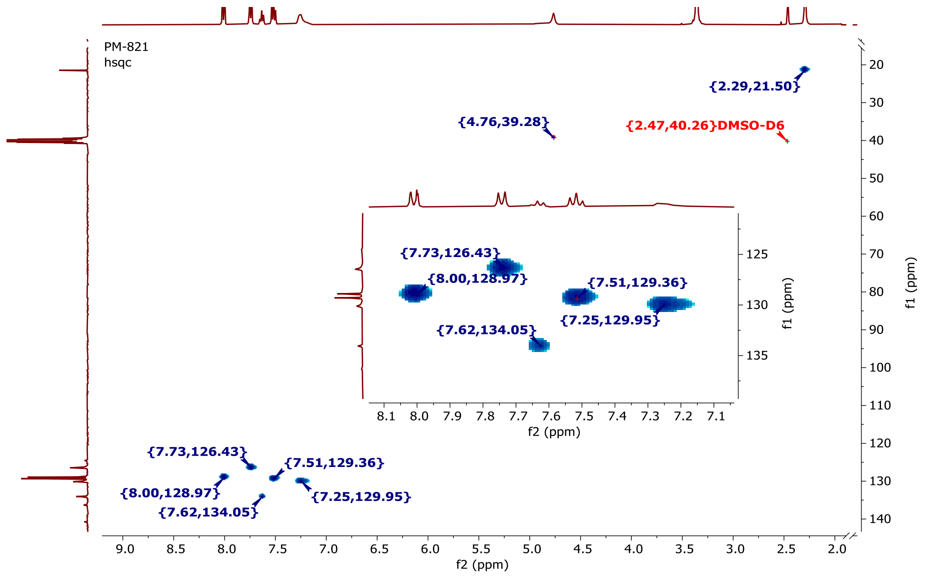

(^{1}H-^{13}C) HMBC spectrum of compound **14a**

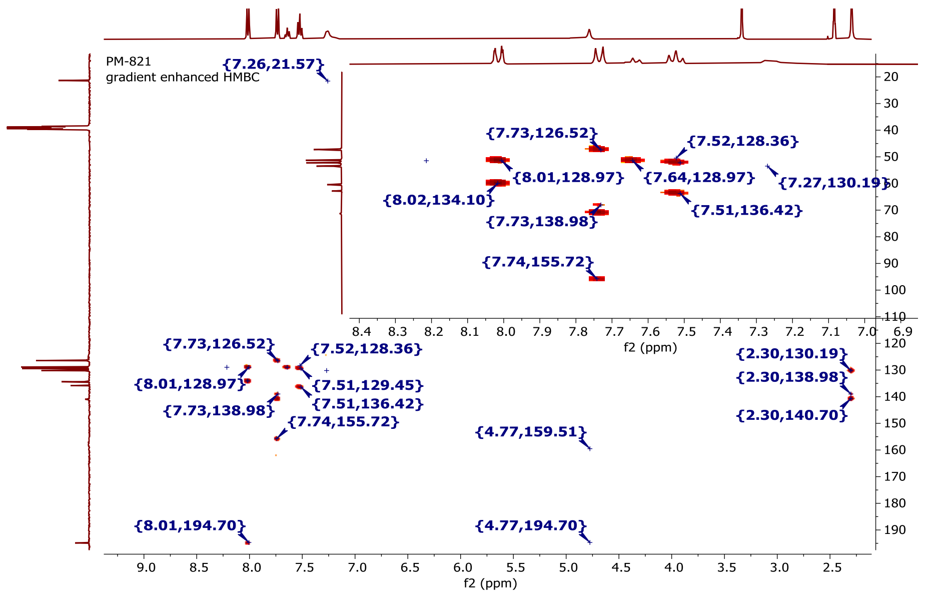

(^{19}F-^{13}C) HMBC spectrum of compound **14a**

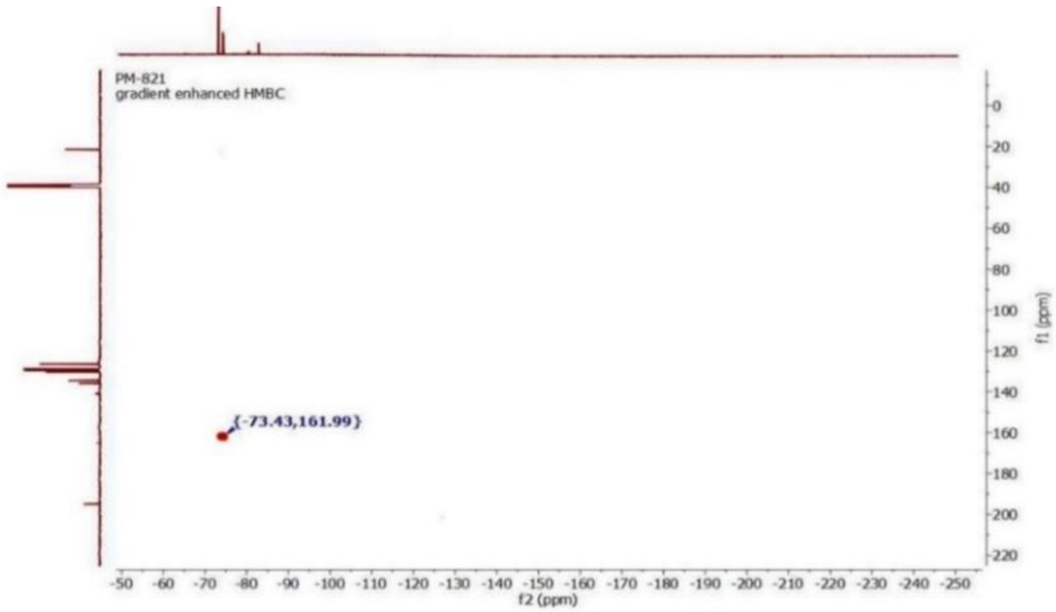

1-Trifluoroacetyl-3-(4-tolyl)-5-((2-oxo-2-(4-fluorophenyl)ethyl)thio)-1,2,4-triazole 14b

^{1}H of 14b

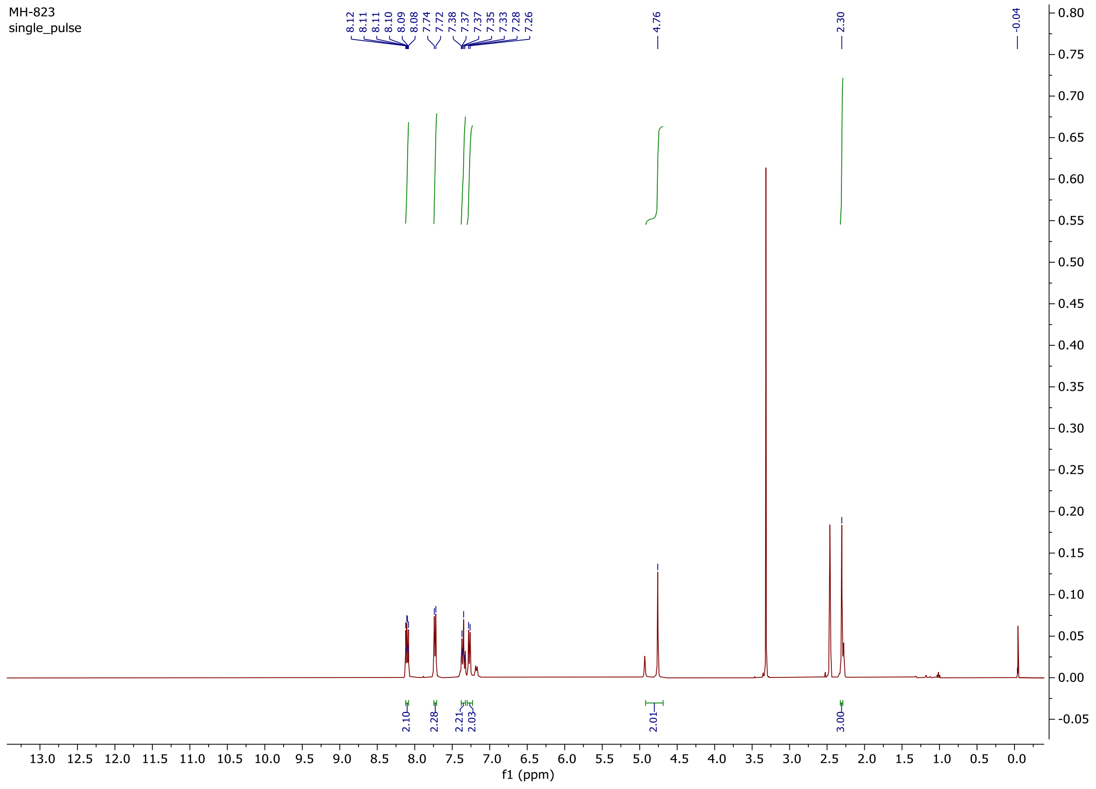

^{13}C of 14b

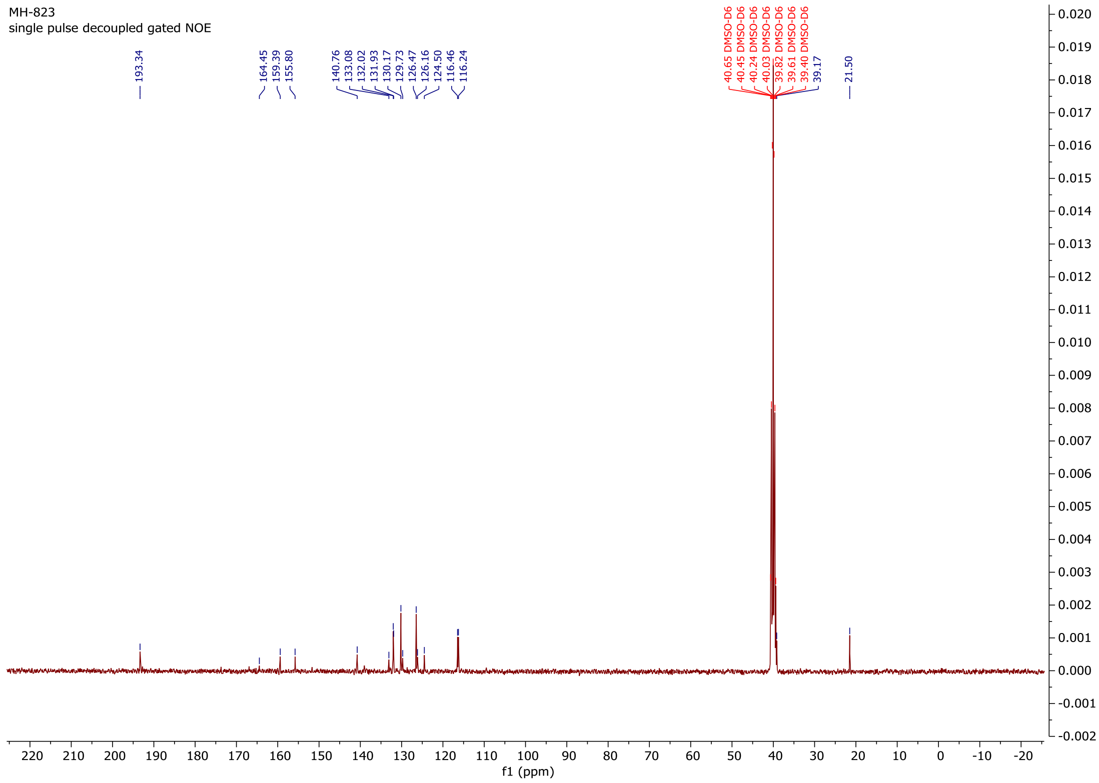

1-Trifluoroacetyl-3-(4-tolyl)-5-((2-oxo-2-(4-chlorophenyl)ethyl)thio)-1,2,4-triazole 14c

^{1}H of 14c

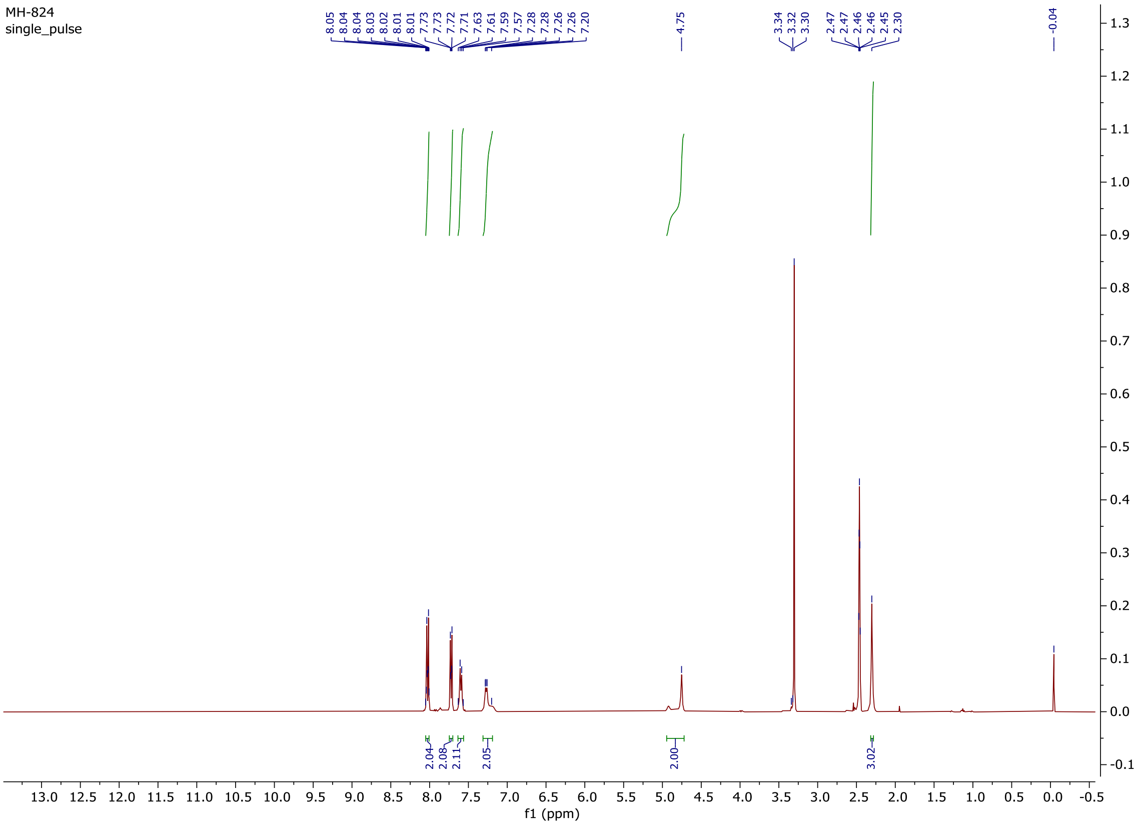

^{13}C of 14c

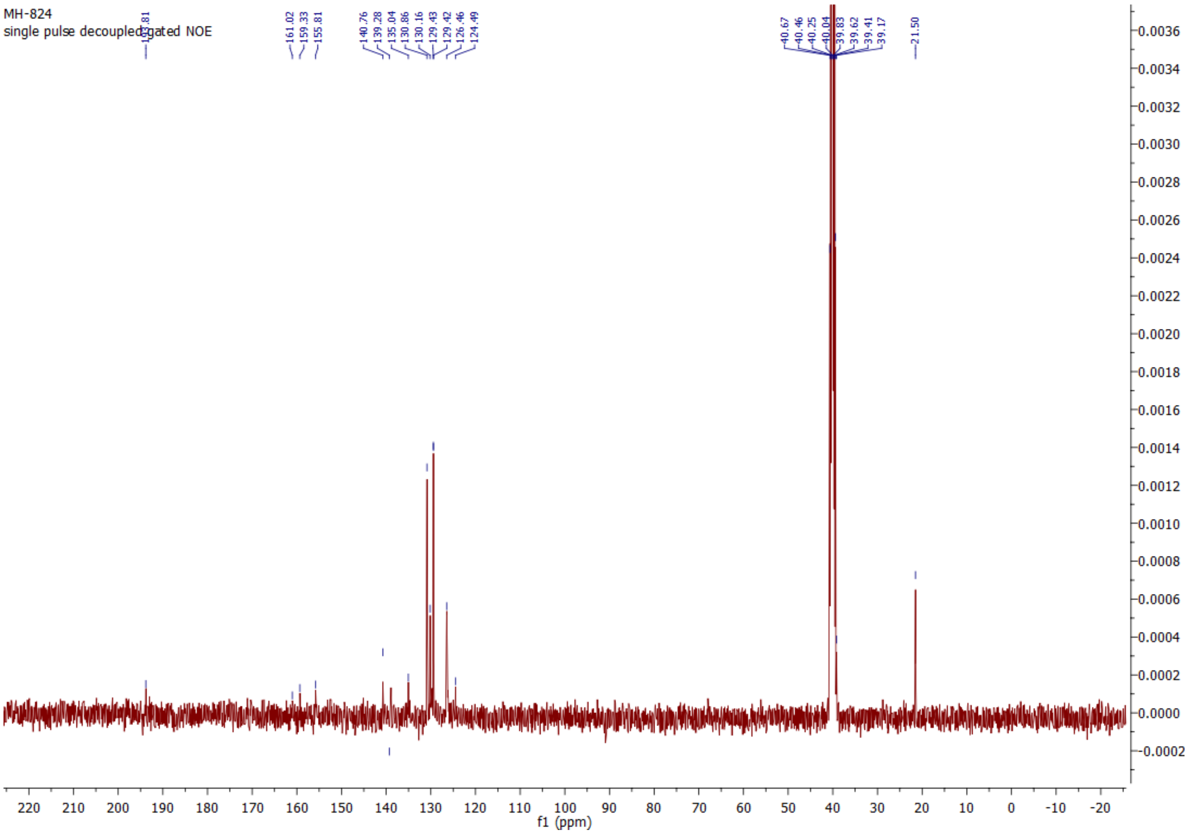

1-Trifluoroacetyl-3-(4-tolyl)-5-((2-oxo-2-(thienyl)ethyl)thio)-1,2,4-triazole 14d

^{1}H of 14d

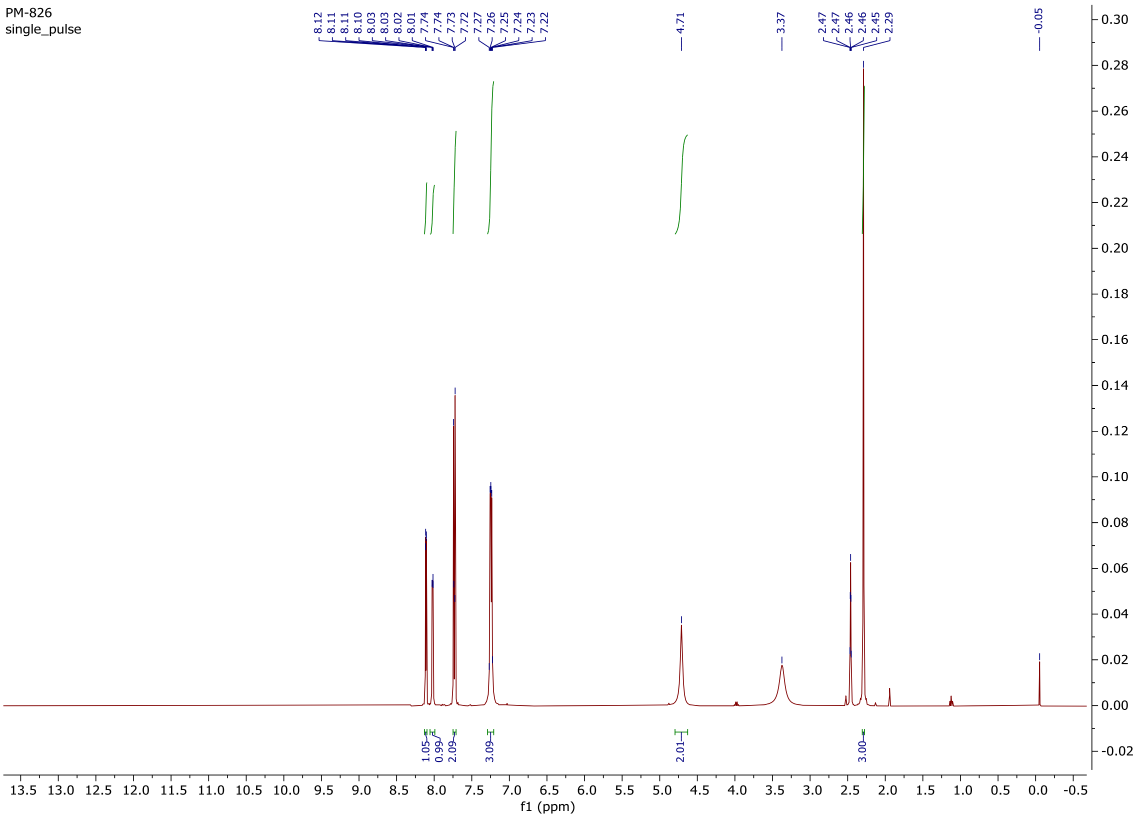

^{13}C of 14d

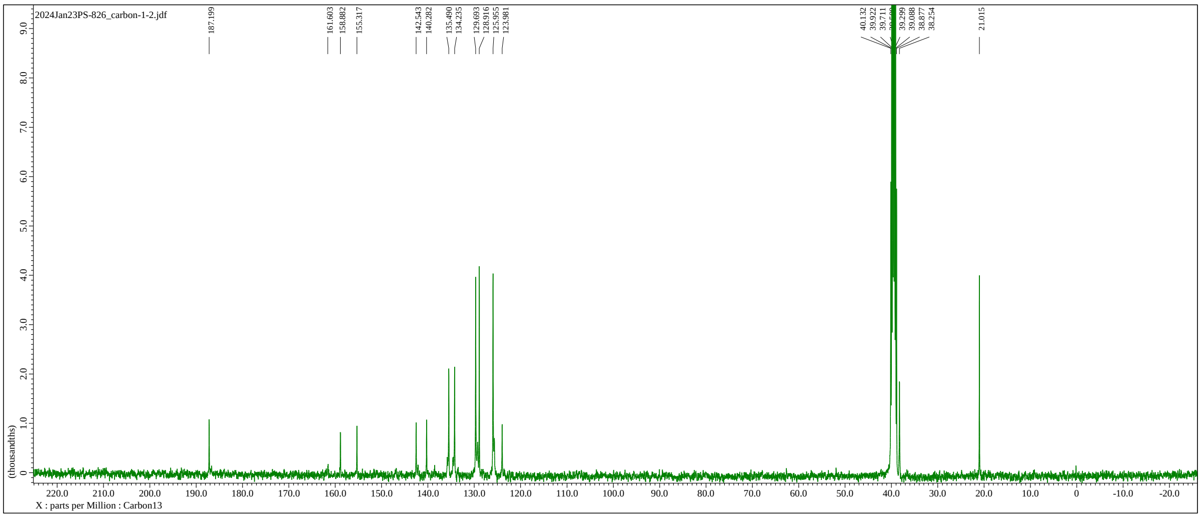

1-Trifluoroacetyl-3-phenyl)-5-((2-oxo-2-(4-fluorophenyl)ethyl)thio)-1,2,4-triazole 14e

[1]H of 14e

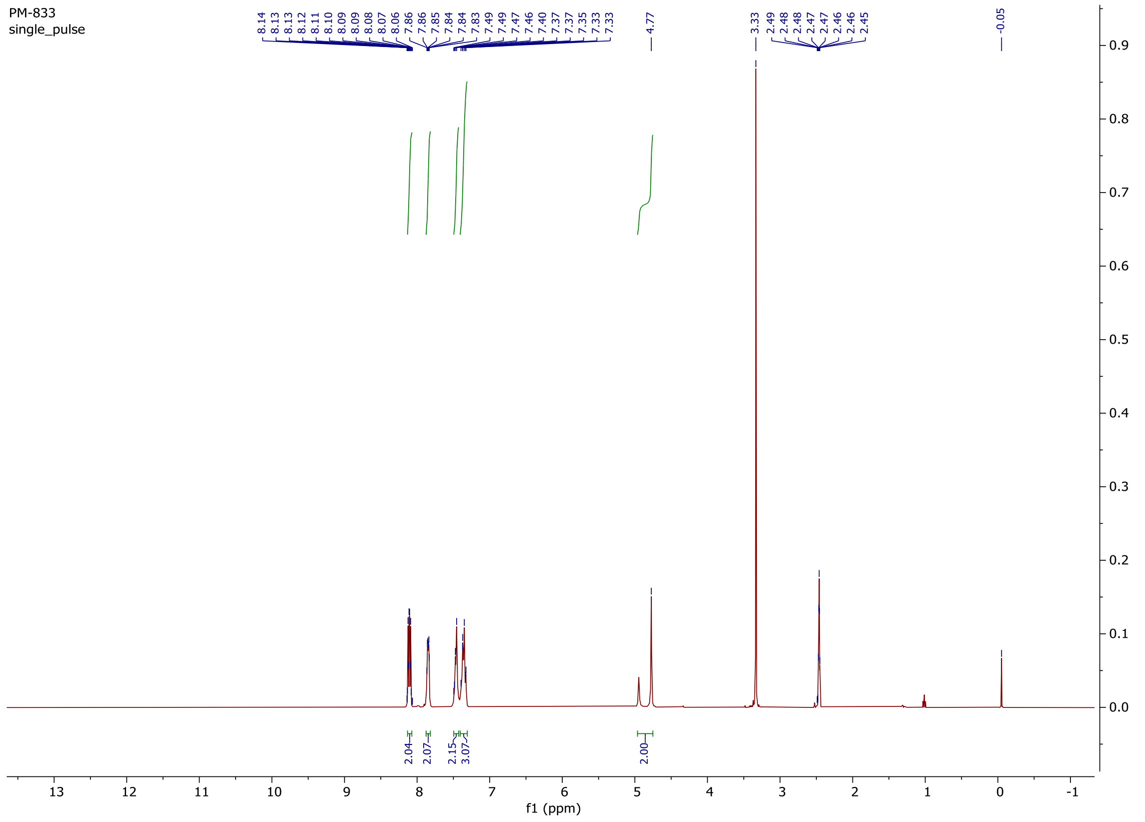

[13]C of 14e

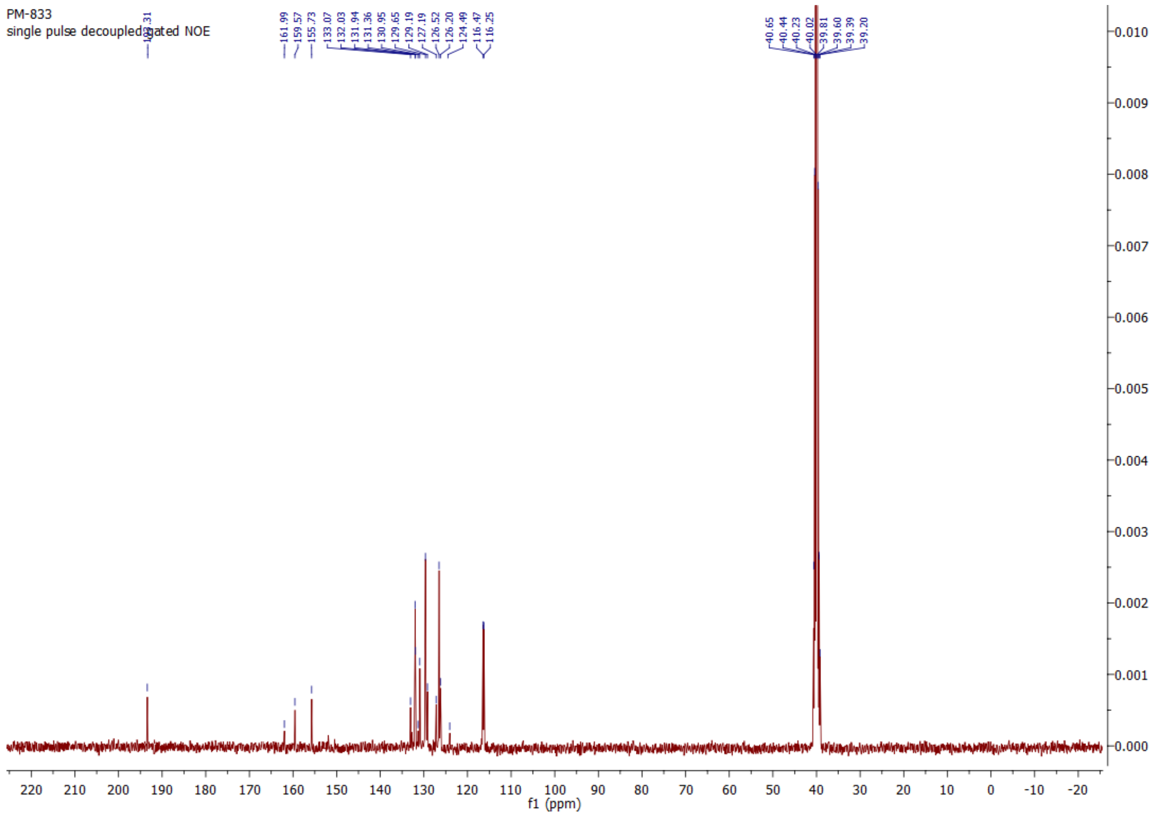

1-Trifluoroacetyl-3-phenyl-5-((2-oxo-2-(4-chlorophenyl)ethyl)thio)-1,2,4-triazole 14f

^{1}H of 14f

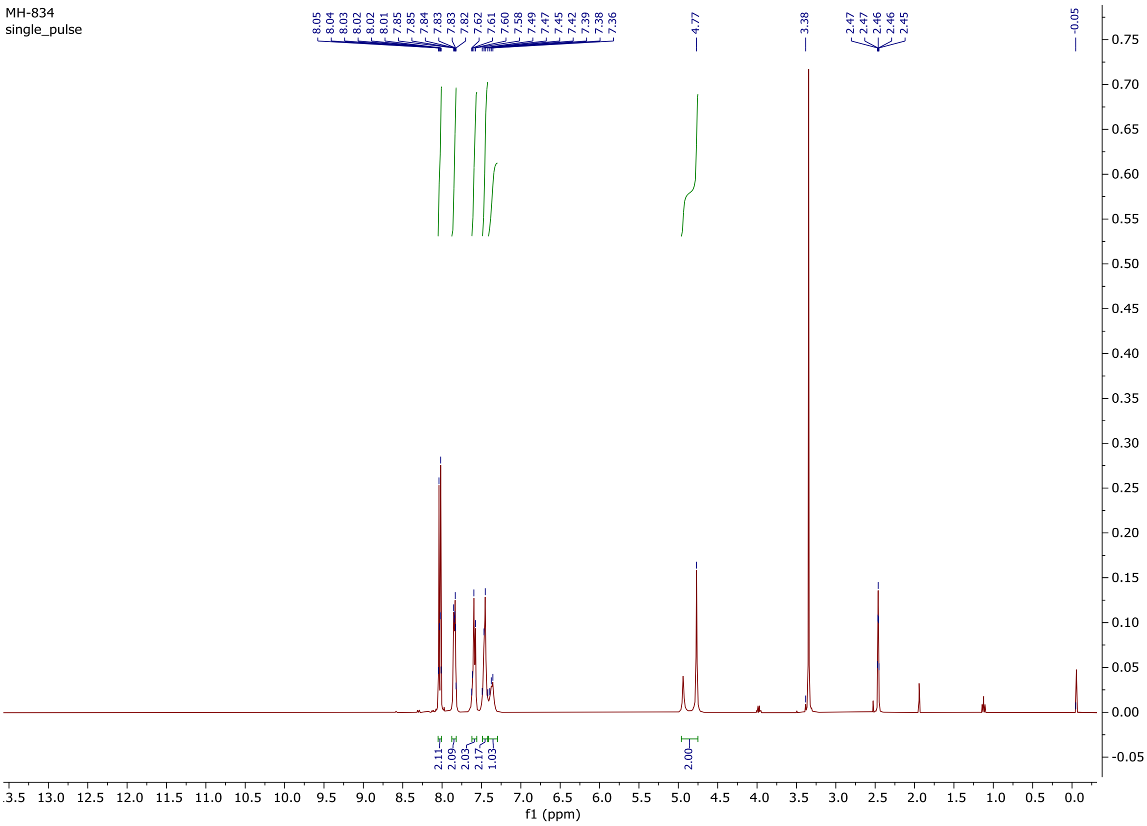

^{13}C of 14f

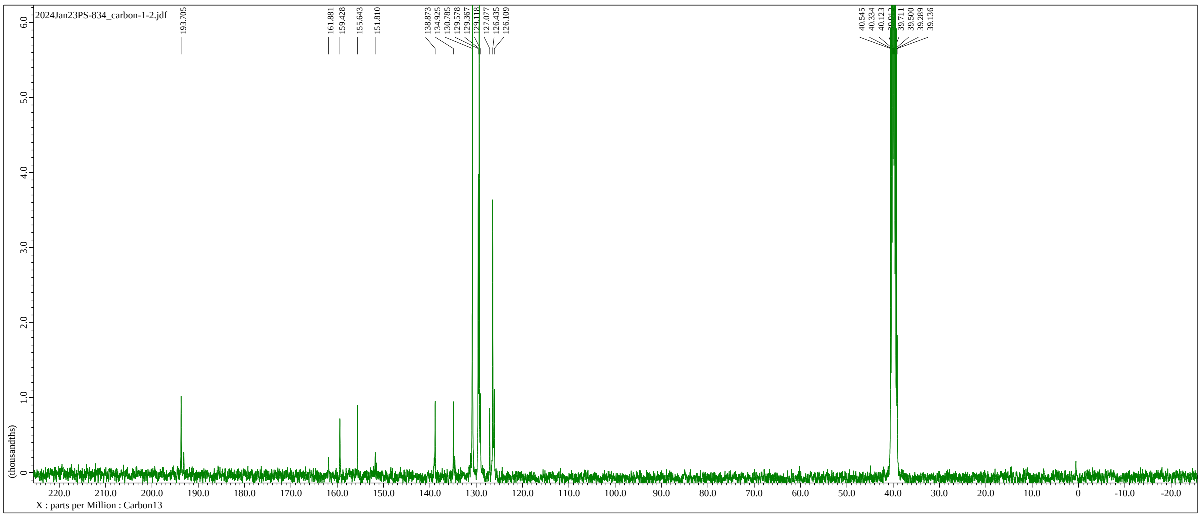

1-Trifluoroacetyl-3-(4-methoxyphenyl)-5-((2-oxo-2-phenylethyl)thio)-1,2,4-triazole 14g

^{1}H of 14g

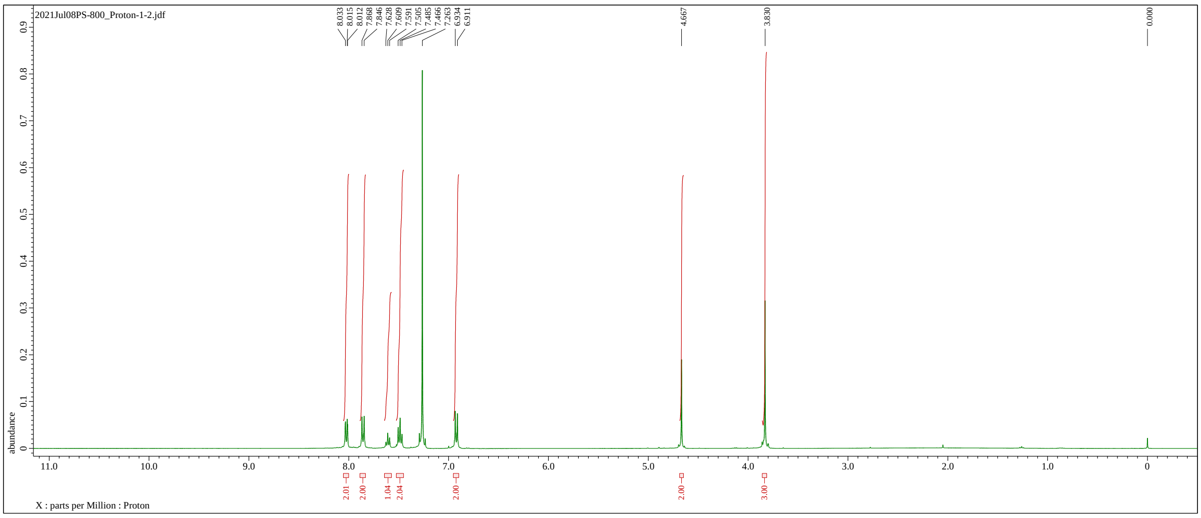

^{13}C of 14g

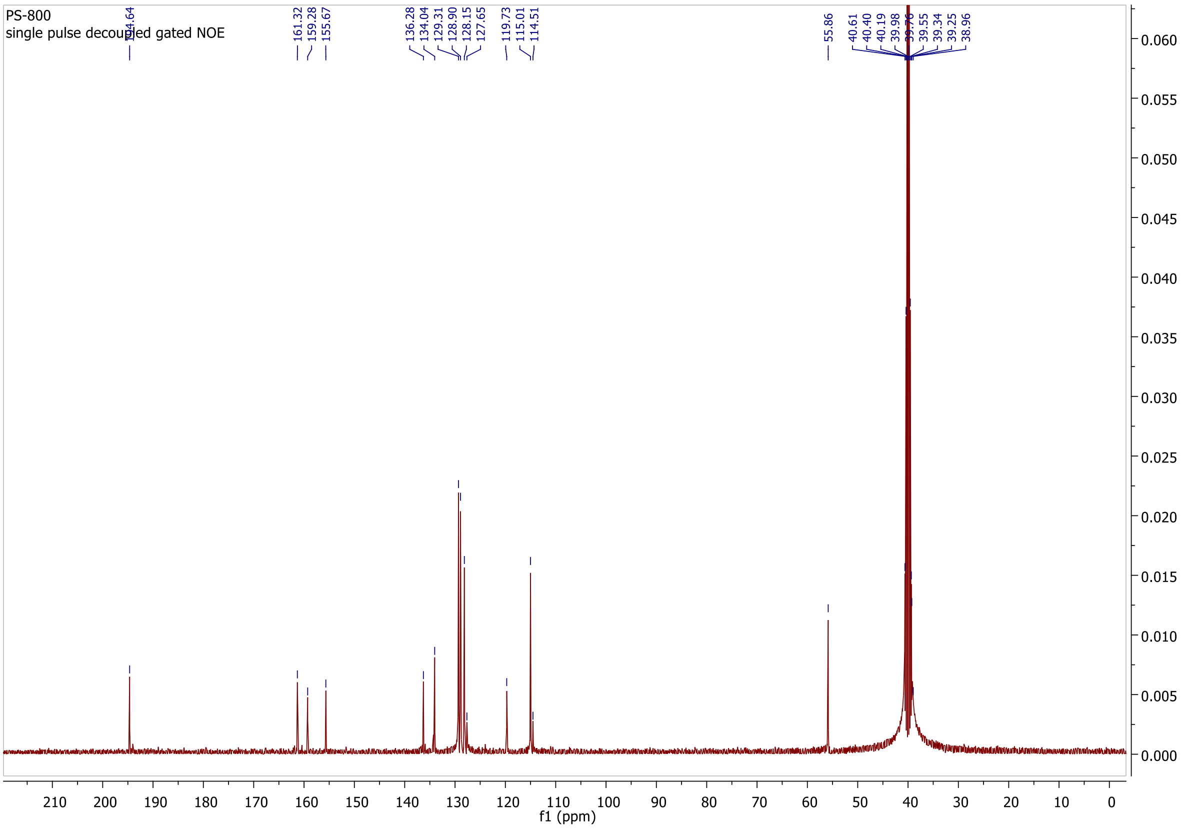

1-Trifluoroacetyl-3-(4-methoxyphenyl)-5-((2-oxo-2-(4-fluorophenyl)ethyl)thio)-1,2,4-triazole 14h

^{1}H of 14h

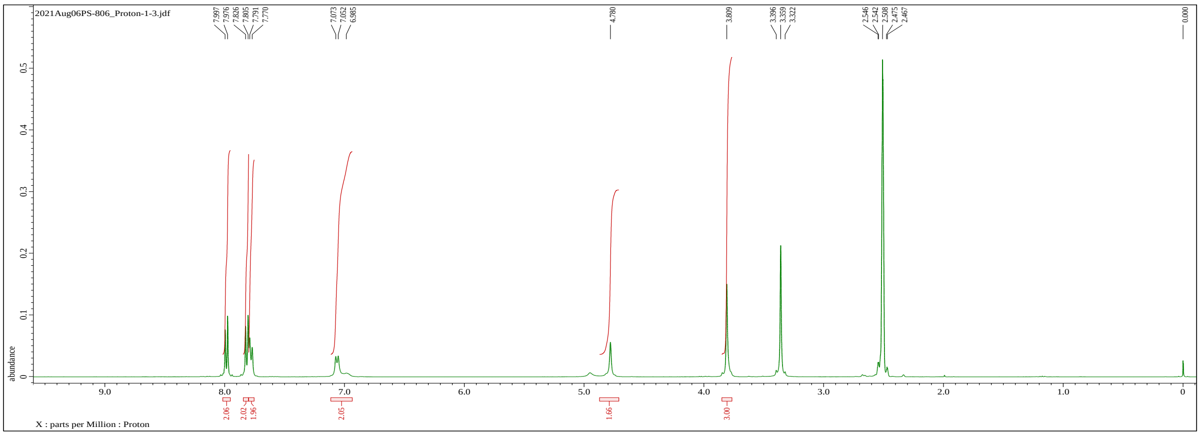

^{13}C of 14h

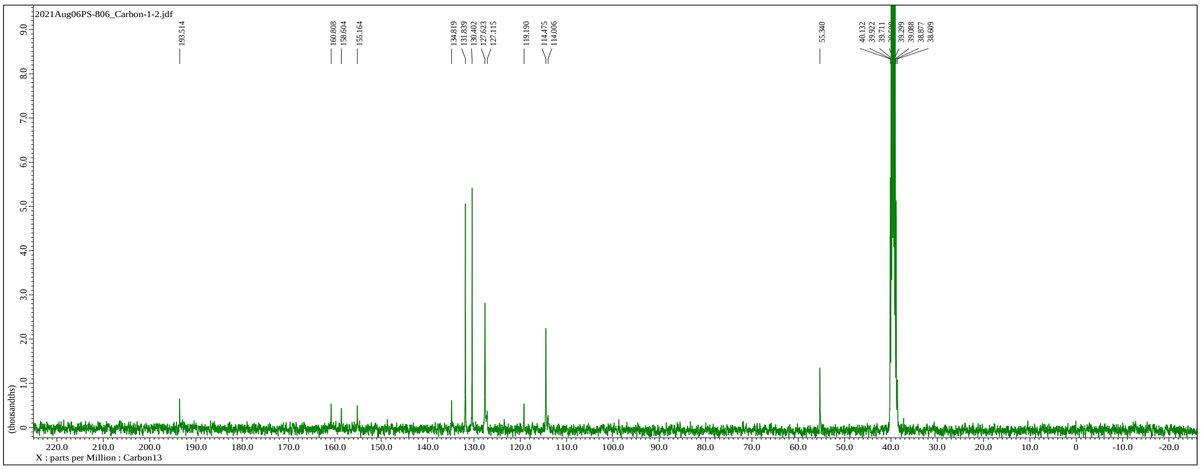

1-Trifluoroacetyl-3-(4-methoxyphenyl)-5-((2-oxo-2-(4-bromophenyl)ethyl)thio)-1,2,4-triazole 14i

^{1}H of 14i

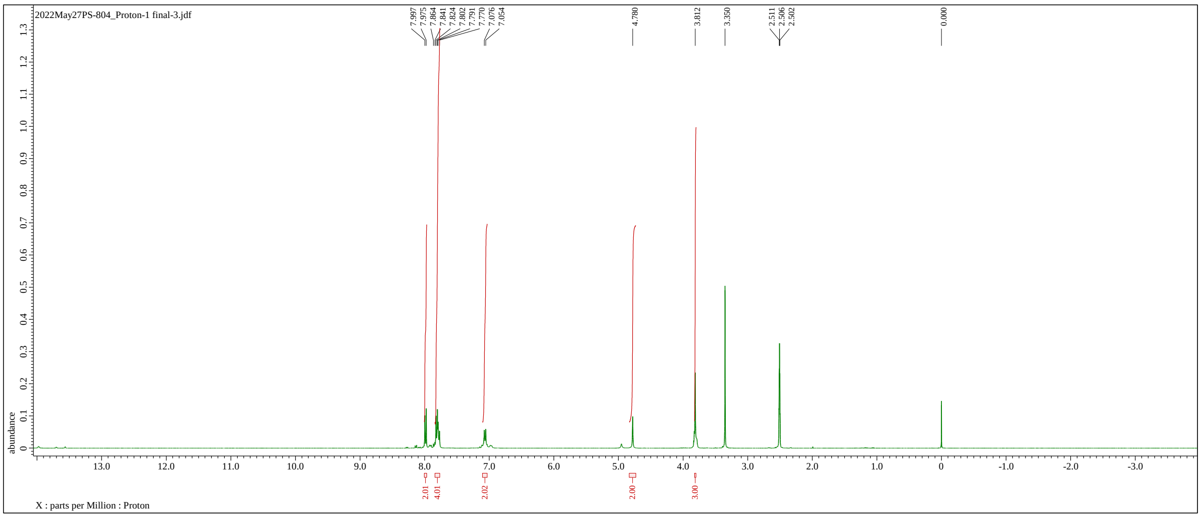

^{13}C of 14i

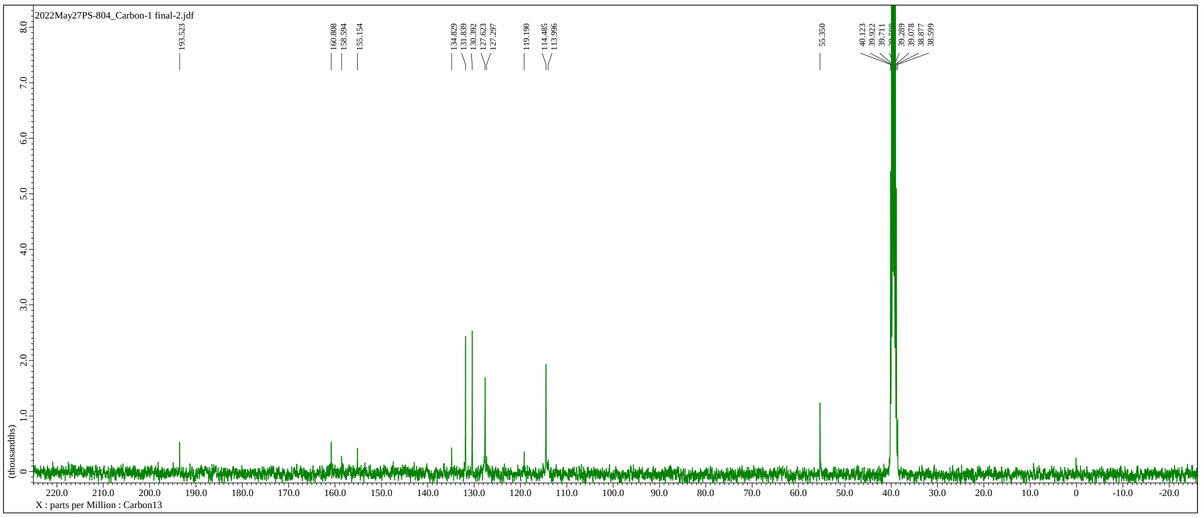

1-Trifluoroacetyl-3-(4-methoxyphenyl)-5-((2-oxo-2-(2,4-dichlorophenyl)ethyl)thio)-1,2,4-triazole 14j

^{1}H of 14j

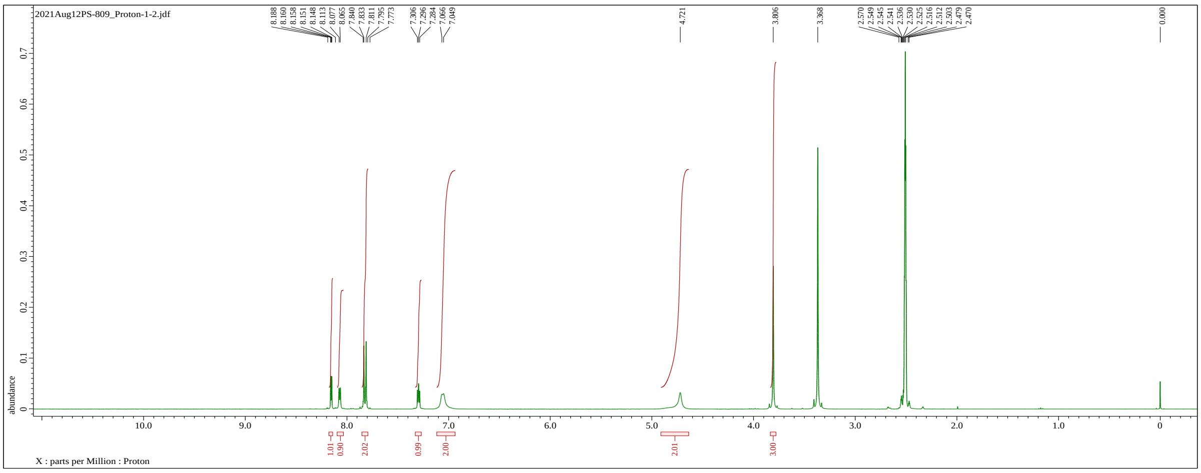

^{13}C of 14j

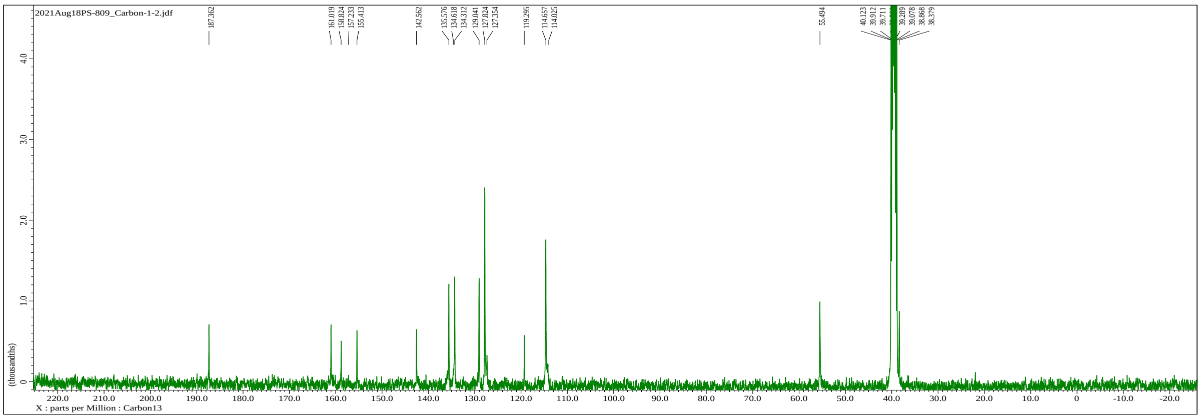

1-Trifluoroacetyl-3-(4-methoxyphenyl)-5-((2-oxo-2-(4-methoxyphenyl)ethyl)thio)-1,2,4-triazole 14k

^{1}H of 14k

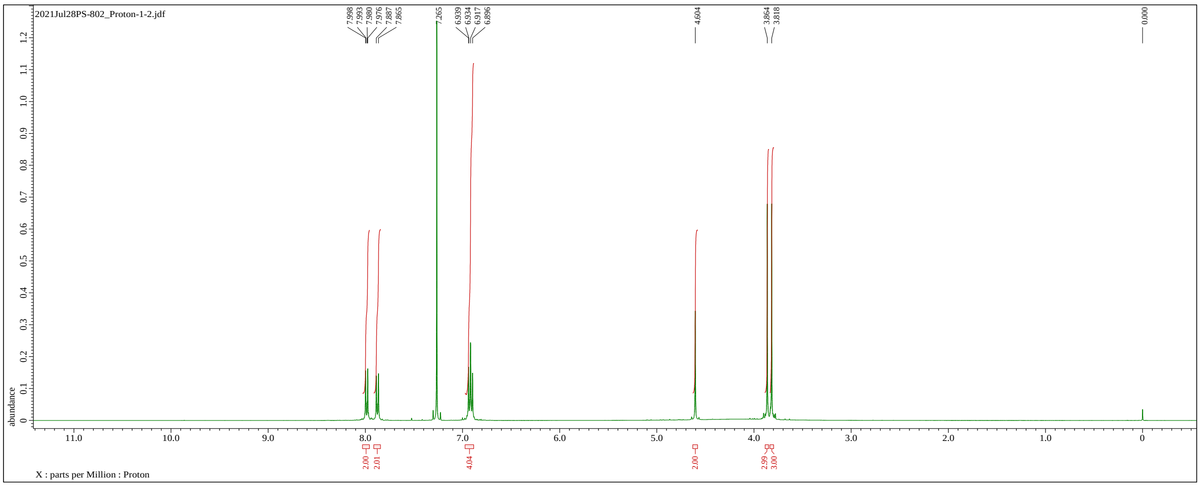

^{13}C of 14k

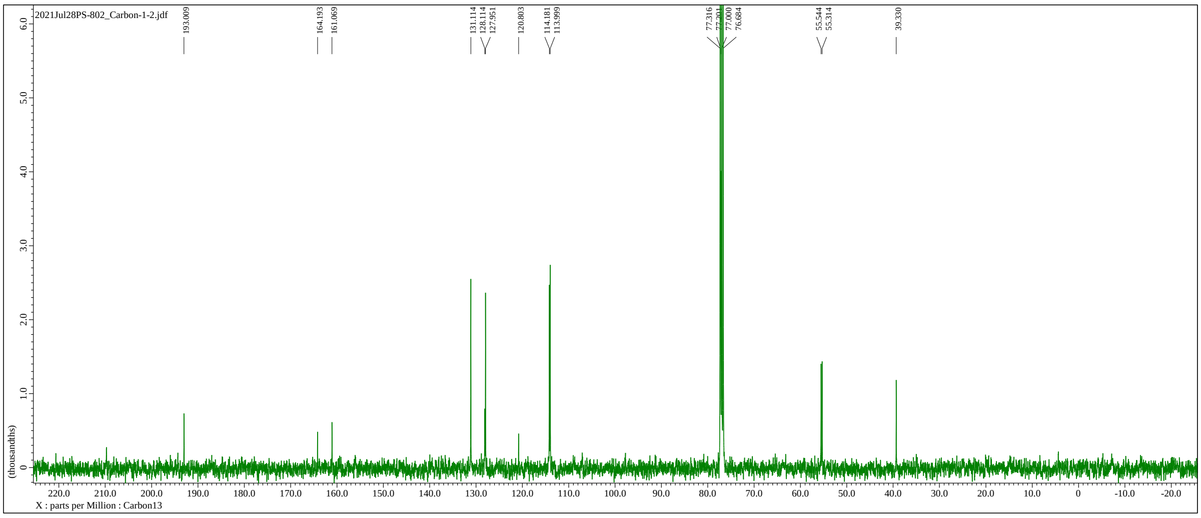

1-Trifluoroacetyl-3-(4-methoxyphenyl)-5-((2-oxo-2-thienylethyl)thio)-1,2,4-triazole 14l

^{1}H of 14l

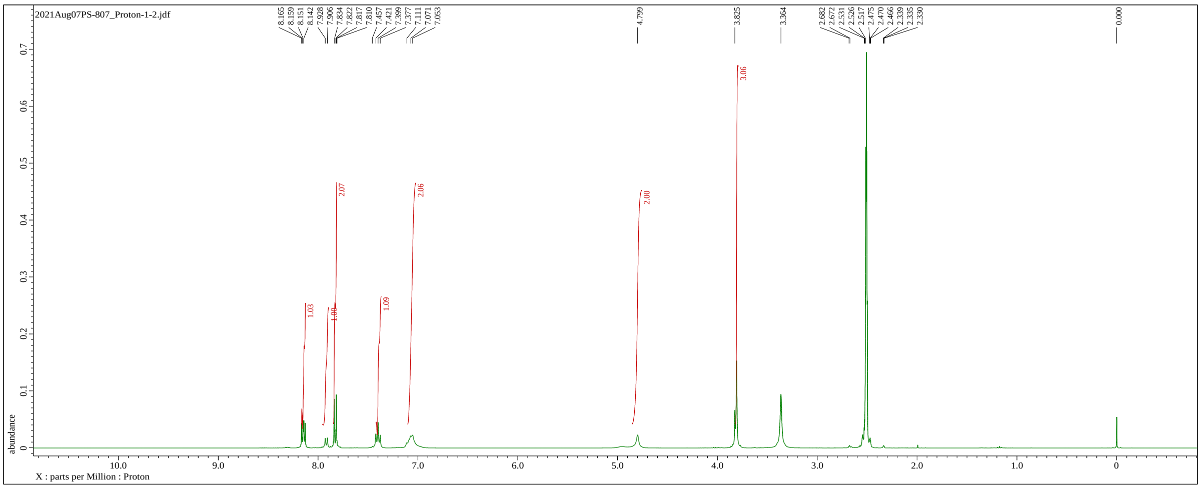

^{13}C of 14l

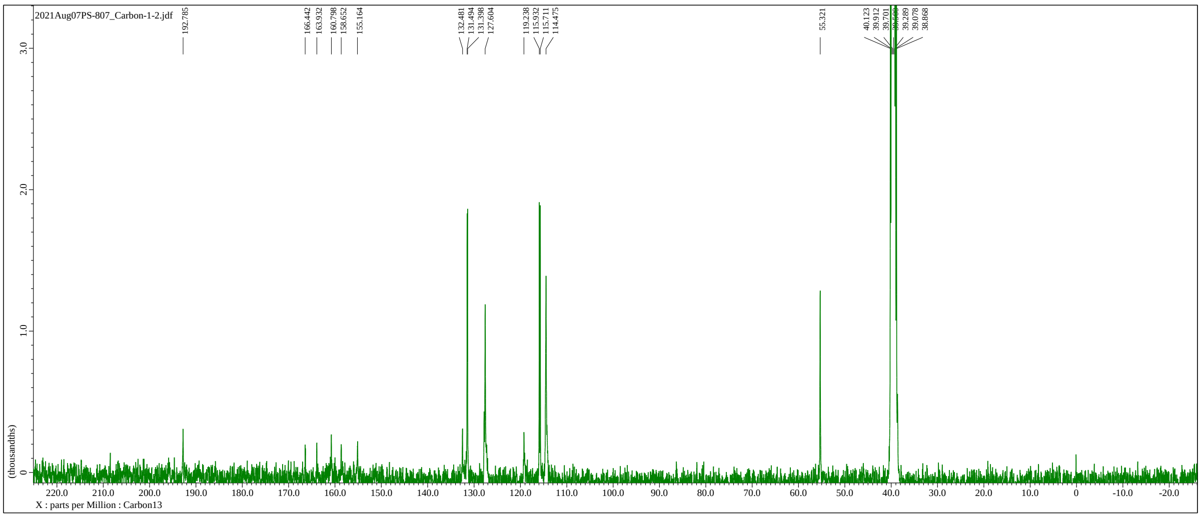

1-Trifluoroacetyl-3-(4-methoxyphenyl)-5-((2-oxo-2-(4-tolyl)ethyl)thio)-1,2,4-triazole 14m

^{1}H of 14m

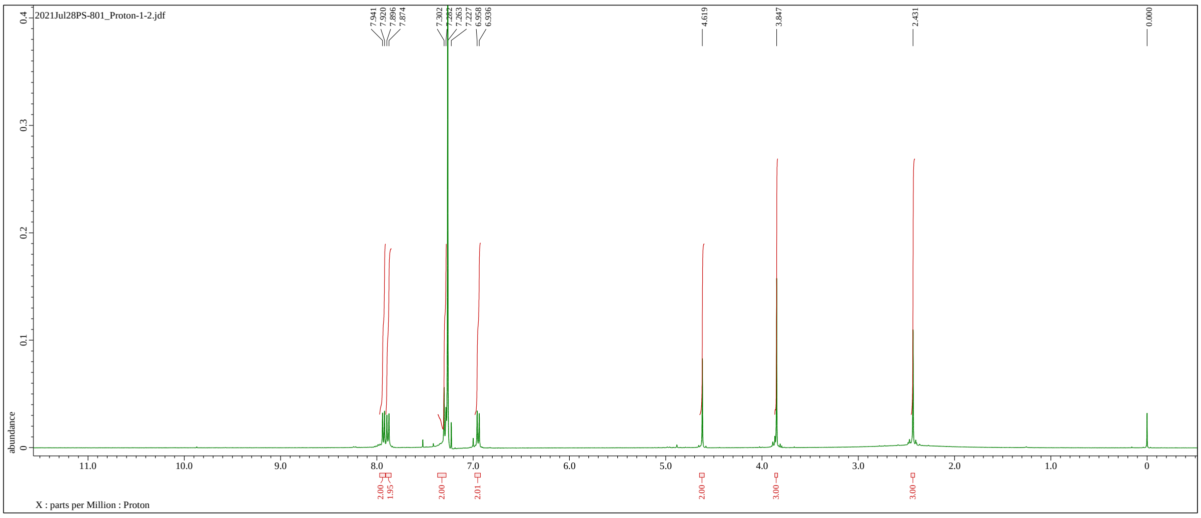

[13]C of 14m

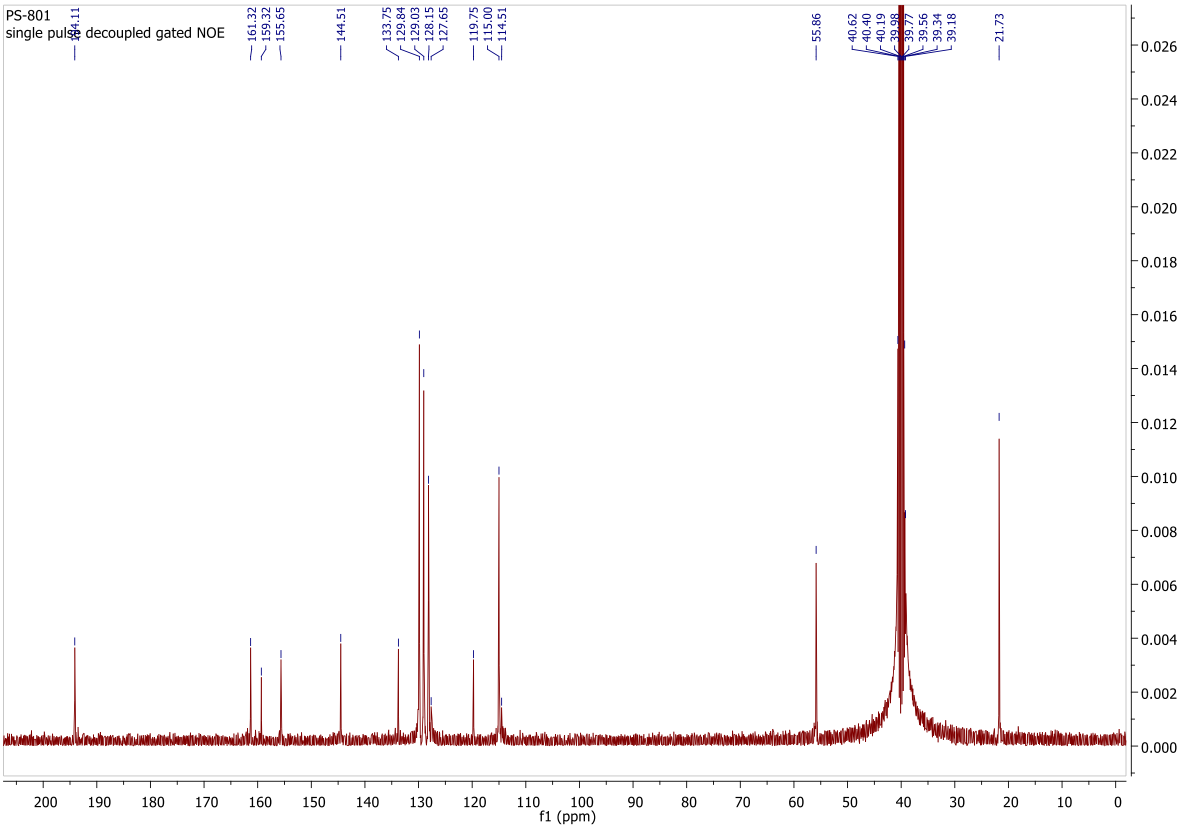

1-Trifluoroacetyl-3-(4-chlorophenyl)-5-((2-oxo-2-(4-bromophenyl)ethyl)thio)-1,2,4-triazole 14n

[1]H of 14n

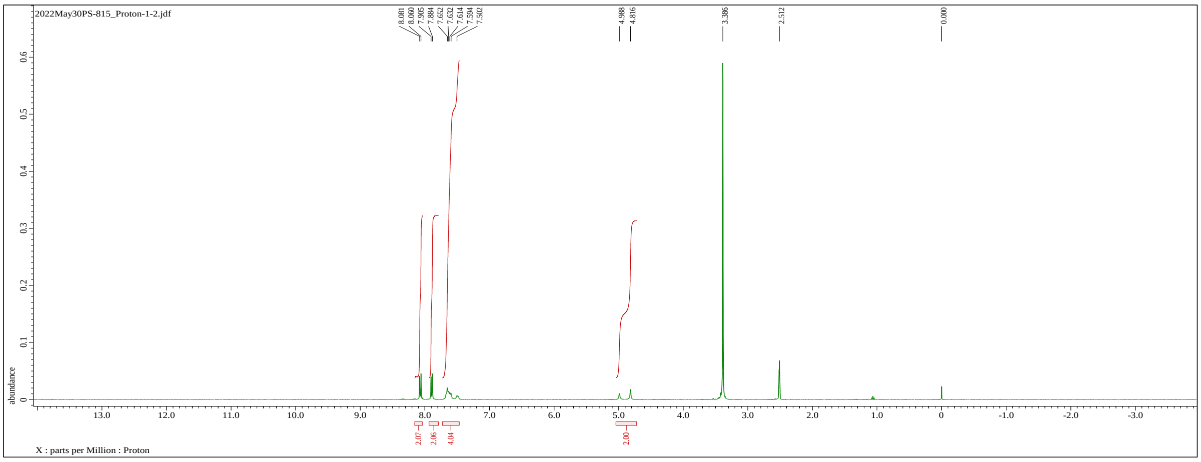

^{13}C of 14n

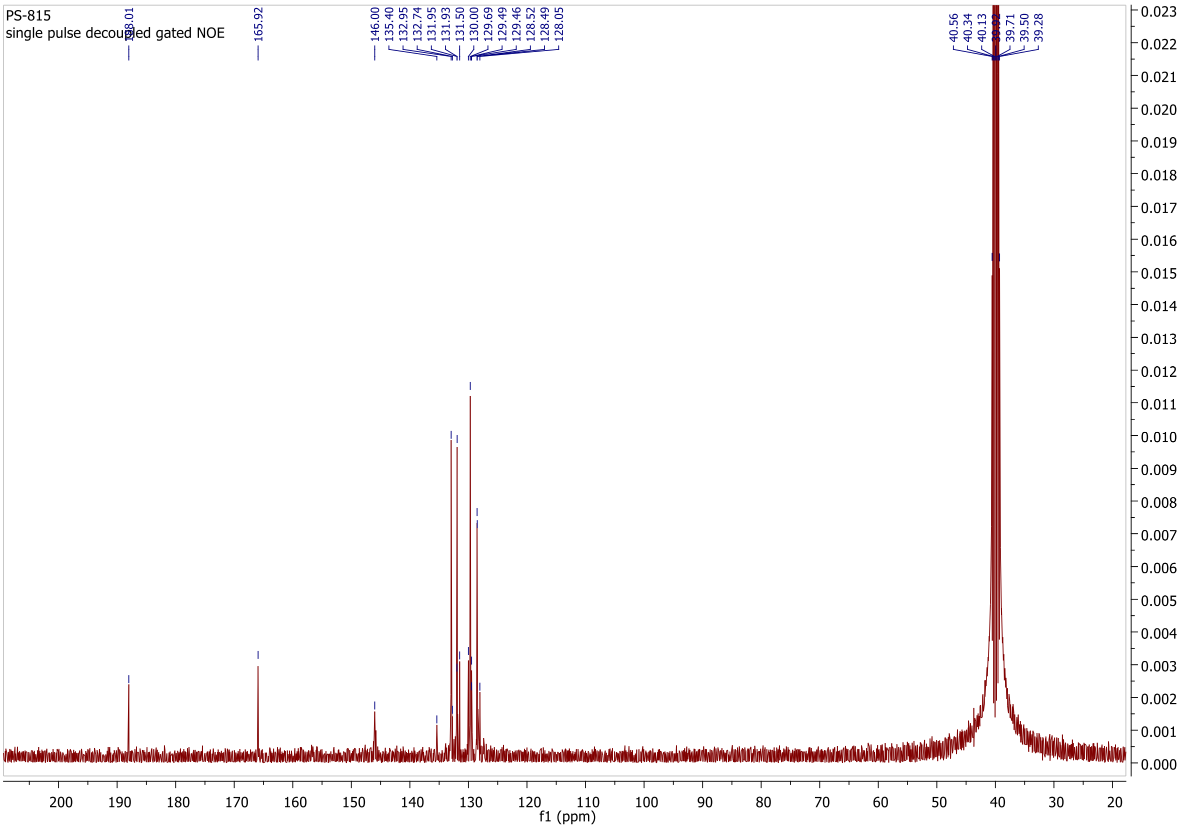

1-Trifluoroacetyl-3-(4-chlorophenyl)-5-((2-oxo-2-thienylethyl)thio)-1,2,4-triazole 14o

^{1}H of 14o

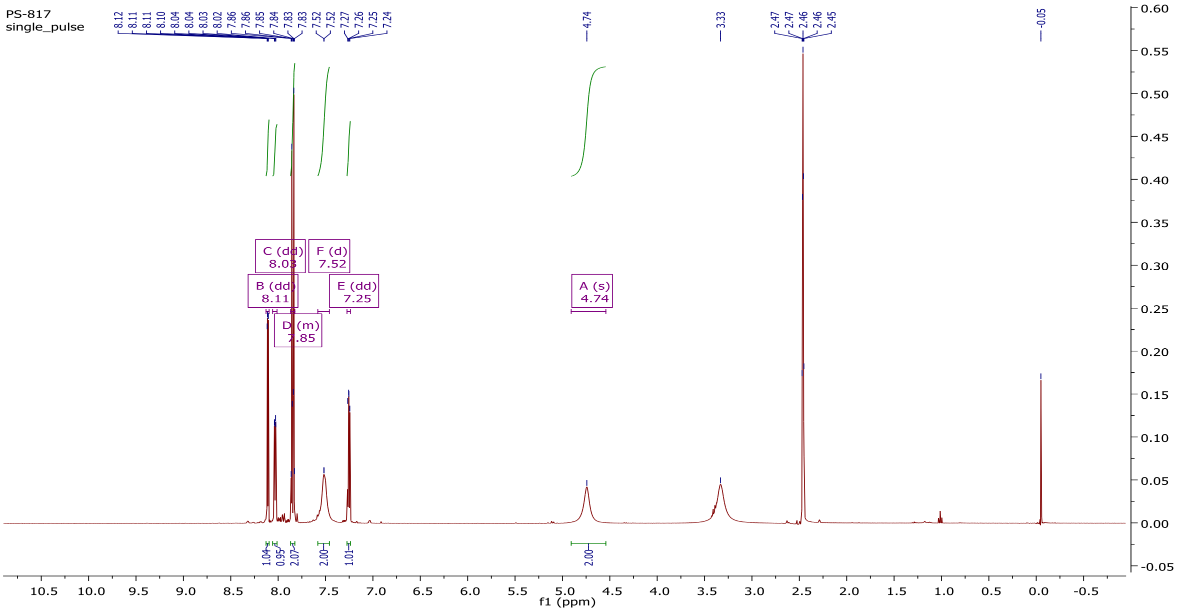

^{13}C of 14o

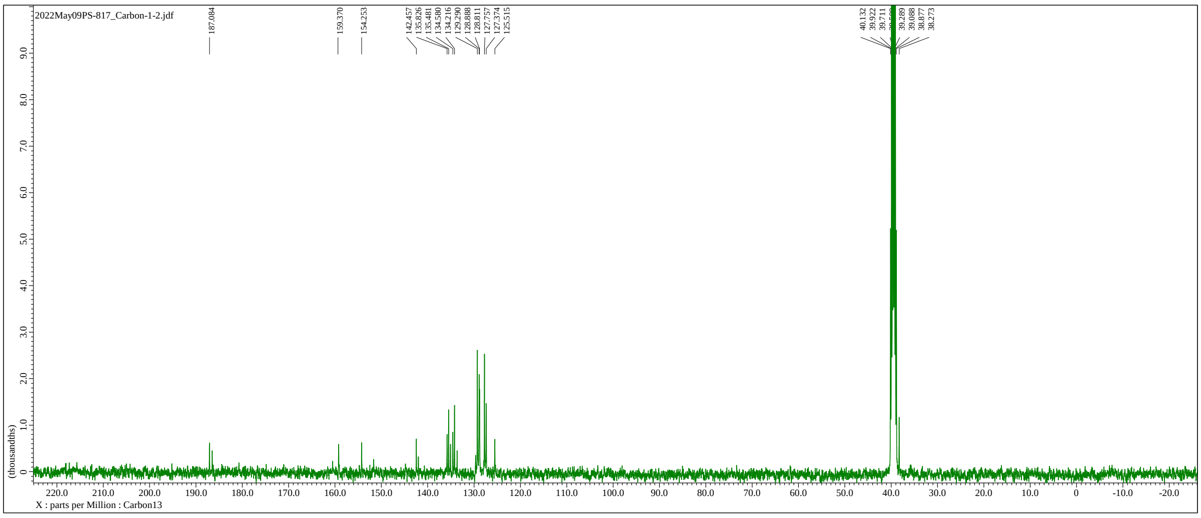

2022May09PS-817_Carbon-1-2.jdf
187.084
159.370
154.253
142.457
135.826
135.481
134.580
134.216
129.290
128.888
128.811
127.757
127.374
125.515
40.132
39.922
39.711
39.289
39.088
38.877
38.273
(thousandths)
X : parts per Million : Carbon13
